# Exo-Geoscience Perspectives Beyond Habitability

**DOI:** 10.1007/s11214-026-01265-y

**Published:** 2026-01-14

**Authors:** Tilman Spohn, Aki Roberge, M. J. Way, João C. Duarte, Francesca Miozzi, Philipp Baumeister, Paul Byrne, Charles H. Lineweaver

**Affiliations:** 1 Institute of Space Research, German Aerospace Center (DLR), Berlin, Germany; 2 Astrophysics Division, NASA Goddard Space Flight Center, Greenbelt, USA; 3 NASA Goddard Institute for Space Studies, New York, USA; 4 Department of Physics and Astronomy, Theoretical Astrophysics, Uppsala, Sweden; 5 Instituto Dom Luiz (IDL), Faculty of Sciences, University of Lisbon, Lisbon, Portugal; 6 Earth and Planets Laboratory, Carnegie Institution for Science, Washington, DC USA; 7 now at: Department of Earth and Planetary Sciences, ETH Zürich, Zürich, Switzerland; 8 and Department of Earth and Environmental Sciences, University of Pavia, Pavia, Italy; 9 Department of Earth Sciences, FU Berlin, Berlin, Germany; 10 Department of Earth, Environmental, and Planetary Sciences, Washington University, St Louis, USA; 11 Planetary Science Institute, The Australian National University, Canberra, Australia

**Keywords:** Rocky exoplanets, Planetary interiors, Atmosphere and oceans, Magnetic fields, Habitability, Life detection

## Abstract

This article reviews the emerging field of exo-geoscience, focusing on the geological and geophysical processes thought to influence the evolution and (eu)habitability of rocky exoplanets. We examine the possible roles of planetary interiors, tectonic regimes, continental coverage, volatile cycling, magnetic fields, and atmospheric composition and evolution in shaping long-term climate stability and biospheric potential. Comparisons with Earth and other planets in the Solar System highlight the diversity of planetary conditions and the rarity of conditions relevant to life. We also discuss contingency and convergence in planetary and biological evolution as they relate to the spread of life in the universe. The observational limits of current and planned missions are assessed, emphasizing the need for models that connect internal dynamics to detectable atmospheric and surface signatures as well as the need for laboratory measurements of planetary properties under a wide range of conditions. The large number of exoplanets promises opportunities for empirical and statistical studies of processes that may have occurred earlier in Earth’s history, as well as for the other pathways rocky planets and biospheres may take. Thus, exo-geoscience provides a framework for interpreting exoplanet diversity and refining strategies for detecting life beyond the Solar System.

## Introduction

“Where is everybody?”, Enrico Fermi is reported to have asked Emil Konopinski, Edward Teller, and Herbert York on their way to lunch at the Los Alamos National Laboratory some time in the summer of 1950 (Jones 1985). The question verbalizes what has become known as Fermi’s paradox (e.g., Webb 2015): In an universe of presumably trillions of Earth-like planets, why have we not seen signs of (intelligent) life beyond the Earth? Seventy-five years later, the question still puzzles scientists and the interested public, although the paradox has been discussed numerous times (e.g., Webb 2015). Instead, it has recently been argued on the basis of Bayesian statistics and observational data that our planet may be rare and that the vast majority of formally habitable planets in the galaxy may lack important elements to make them truly habitable and inhabited (e.g., Simpson 2017; Scherf et al. 2024; Apai et al. 2025).

**Fig. 1:**
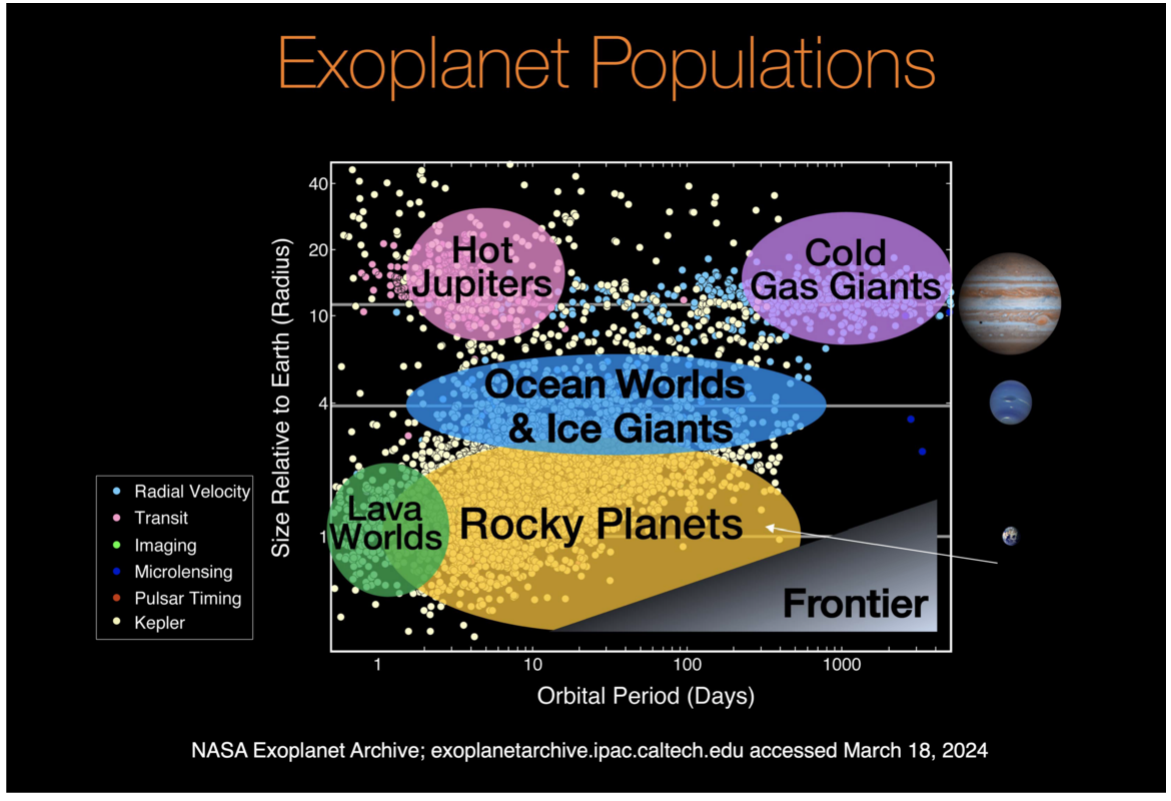
The NASA Exoplanet Archive compiled exoplanet populations. The legend at the left–hand side indicates the detection methods. To date, 6065 exoplanet detections have been confirmed, and 7687 candidates are awaiting confirmation. Of the confirmed planets, 1446 have radii of 2 Earth radii or less, masses between 0.5 and 10 Earth masses, and estimated densities between 3000 and 6000 kg/m^3^. “Rocky planets”, the subject matter of this topical collection, include a wide variety of planets – from sub- to superterran sizes, with or without atmospheres, and with presumably a wide variety of surfaces and interior structures (https://exoplanetarchive.ipac.caltech.edu, retrieved Dec 17, 2025)

Although exoplanet research is justified in its own right and as part of the broader field of comparative planetology, our focus here is on exoplanets as potential hosts of extraterrestrial life. Over 6000 exoplanets have been detected, with another almost 8000 candidates awaiting confirmation (https://exoplanetarchive.ipac.caltech.edu, retrieved Dec 17, 2025, compare Fig. 1). This includes an increasing number of planets that are approximately the size and mass of Earth. A significant number of these planets orbit within their host stars’ habitable zone^1^ and may have the potential for liquid surface water. While the concept of the habitable zone is useful for a first–order assessment of the chance of detecting extraterrestrial life, further refinement is likely needed before investing in costly and sophisticated follow-up observations (e.g., Glaser et al. 2020). More specific planetary physical properties to consider include the balance between land and (deep) ocean surfaces, the availability of water versus nutrients from riverine sources such as phosphorus and nitrogen, signs of thermodynamic disequilibrium (e.g. Schwieterman et al. 2018), and the potential for building-up free oxygen to support more complex life forms. In addition, tectonic modes and magnetic fields may matter. The article by Glaser et al. (2026) in this topical collection introduces a new term to capture these additional factors: “euhabitable”, meaning *truly* habitable. While these planetary properties are difficult to observe remotely with present means (see Sect. 3 below), they may be important for understanding the conditions under which life can form and evolve.

For geoscientists, rocky exoplanets allow for an assessment of how unusual or common Earth is, and motivating scientists to reconsider Earth’s properties in new ways. To quote Shorttle et al. (2021), “…many of Earth’s one-offs – plate tectonics, surface liquid water, a large moon, and life: long considered as ‘Which came first?’ conundrums for geoscientists – may find resolution in the study of exoplanets that possess only a subset of these phenomena.” Eventually, sufficient numbers of exoplanets may be observed at different stages of their evolution to allow for statistically robust conclusions to be drawn. We may also have the chance to observe planets that took different evolutionary paths, whether geological or biological. The search for life beyond the Solar System may even further our understanding of the future of life on Earth, albeit indirectly, as discussed in e.g., Lingam and Balbi (2024, Part IV). For example, we may learn whether complex life persists once it arises or whether such life represents a fleeting moment in a planet’s evolution on geological timescales.

This article is organized into three main sections. The next section discusses what can be inferred from studies of Earth and terrestrial life for exoplanet and life detection studies. Next, we report on upcoming missions to observe rocky planets, with an emphasis on life detection. Lastly, we discuss the interest that geoscientists may have in the diversity of planets. We close by sharing some concluding thoughts on the emerging field of Exo-Geoscience.

## Geoscience Lessons for Exoplanet Science

The Copernican Principle^2^ (e.g., Bondi 1952) and the Principle of Mediocrity^3^ - both widely accepted - form the basis of the argument that the universe should be teeming with life (for a recent review and critical discussion of the mediocrity principle as applied to astrobiology, see Balbi and Lingam 2023 and Lingam and Balbi 2024). However, from the perspective of the Solar System, Earth appears unique as the only planet known to support life. Its combination of geological and astronomical properties may even be rare in the present galaxy, perhaps even in the Universe (e.g., Taylor 1998; Ward and Brownlee 2000; Waltham 2014; Simpson 2017; Scherf et al. 2024). Some characteristics that may be particularly important but rare are:

- the right size (mass) rocky planet in the star’s habitable zone
- plate tectonics and a magnetic field,
- land masses and oceans on the surface,
- a N−O atmosphere with some CO_2_, or simply N+CO_2_
- a large moon stabilizing the rotation axis against obliquity excursions and thus stabilizing climate zones (Laskar et al. 1993), and
- a giant planet like Jupiter guarding against a high impact flux (e.g. Wetherill 1994).

Other properties are more related to the Earth’s place in the galaxy, such as orbiting a G-type star in the galactic habitable zone,^4^ and perhaps early ejection of the Sun from its original stellar nursery (e.g. Adams 2010).

The Rare Earth hypothesis has been criticized for being anthropocentric and overemphasizing the needs of *life-as-we-know-it*. For example, Kasting (2001) and specifically Langmuir and Broeker (2012, p.384) argue that life is an “efficient and natural planetary process”, drawing energy from its environment while increasing the system’s entropy and maximizing entropy production (see also Kleidon and Lorenz 2005). They argue that this process should “occur widely throughout the universe”. Others, such as Southam et al. (2015), argue that water and rock should provide all the ingredients needed for life. Indeed, we may be too focused on life as we know it; to quote (not verbatim) the Greek philosopher Xenophanes (570 BC), “if horses had gods, they would represent them as horses” (see also Feinberg and Shapiro 1980 and Ward 2007). But how can we design a search for life that is completely different from what we know? Without a deep understanding of life in general — how it originates and evolves — and how alien life forms and their basis might differ from those on Earth, it is only natural to search for habitats similar to those on our own planet and the other planets in the Solar System.

Commonly used terms such as habitable – or more recently introduced terms such as urable (conditions that allow life to begin; Deamer et al. 2022), euhabitable (truly habitable; Glaser et al. 2026, this topical collection), and fecundity (suitable to support life with technological intelligence;^5^ Simpson 2017) – thus mostly reflect life as we know it (see also Apai et al. 2025 for a discussion of relevant terminology). One open question is how likely it is that a urable and (eu)habitable planet, or even a planet with fecundity, would actually be inhabited. As Deamer et al. (2022) argue, a urable planet would almost certainly exhibit abiogenesis. Due to the proximity of the conditions for urability and euhabitability, it is reasonable to suppose that such a planet would have a high probability of being (or having been) inhabited. However, note that an (eu)habitable planet can evolve away from urability because the latter requires an anoxic environment.^6^ Thus, Earth’s surface today – except for certain anoxic niches – would not qualify as urable (see Deamer et al. 2022 and Lineweaver and Chopra 2012 for discussions). This observation let Lineweaver and Chopra (2025) to propose that life must escape a “bottleneck” before it can coevolve with the planet.

Many features of the present-day Earth (ignoring anthropogenic features) can be linked to the biosphere in one way or another. These features include the composition of the atmosphere (e.g., Grenfell 2017), oceans (e.g., Langmuir and Broeker 2012), and soil (e.g., Retallack 2015), as well as processes like plate tectonics, continental growth (e.g., Rosing et al. 2006; Retallack 2015; Spencer 2022; Höning and Spohn 2023; Stern and Gerya 2024), and mountain building (e.g., Parnell and Brolly 2021), which have all been linked to bioactivity. Microbial activity, moreover, drives Earth’s biogeochemical cycles and promotes the oxygenation of the planet (Falkowski et al. 2008). Consequently, Zuluaga et al. (2014) have proposed the terms “abiotic” and “inhabited habitable zones” to acknowledge that physical parameters used to delineate the boundaries of a circumstellar habitable zone may be modified by life. From a chemical perspective, life – as Langmuir and Broeker (2012) noted – is a complement of the solid Earth.

### The Earth and the Solar System Planets as Reference Cases: Properties and Processes

Earth is the 3rd planet from the Sun in the Solar System. The Earth in comparison with Solar System planets and moons and known exoplanets is the subject of the article by Byrne et al. (2026) in this topical collection. The mass of Earth is sufficient to gravitationally bind a substantial N–O–dominated atmosphere (see Steinmeyer et al. 2026 and Kubyshkina et al. 2026, this topical collection). Venus, of similar mass, binds a much heavier, CO_2_-dominated atmosphere with only minute amounts of molecular oxygen (e.g., Baines et al. 2013). It is widely believed that the difference in atmospheric composition between the two neighboring planets is caused by differences in their early evolution (e.g. Hamano et al. 2013; Lebrun et al. 2013) or was driven by a yet to–be–determined catastrophic climate change on Venus sometime in its history (Way and Del Genio 2020; Way et al. 2022). Mars, about half the size of Earth and 1/10th the mass, could not sustain a habitable atmosphere long enough for a sustained surface life cycle (Wordsworth 2016), so any remaining biosphere would be subterranean.

#### Interior Properties and Processes

##### Interior Structure

**Fig. 2:**
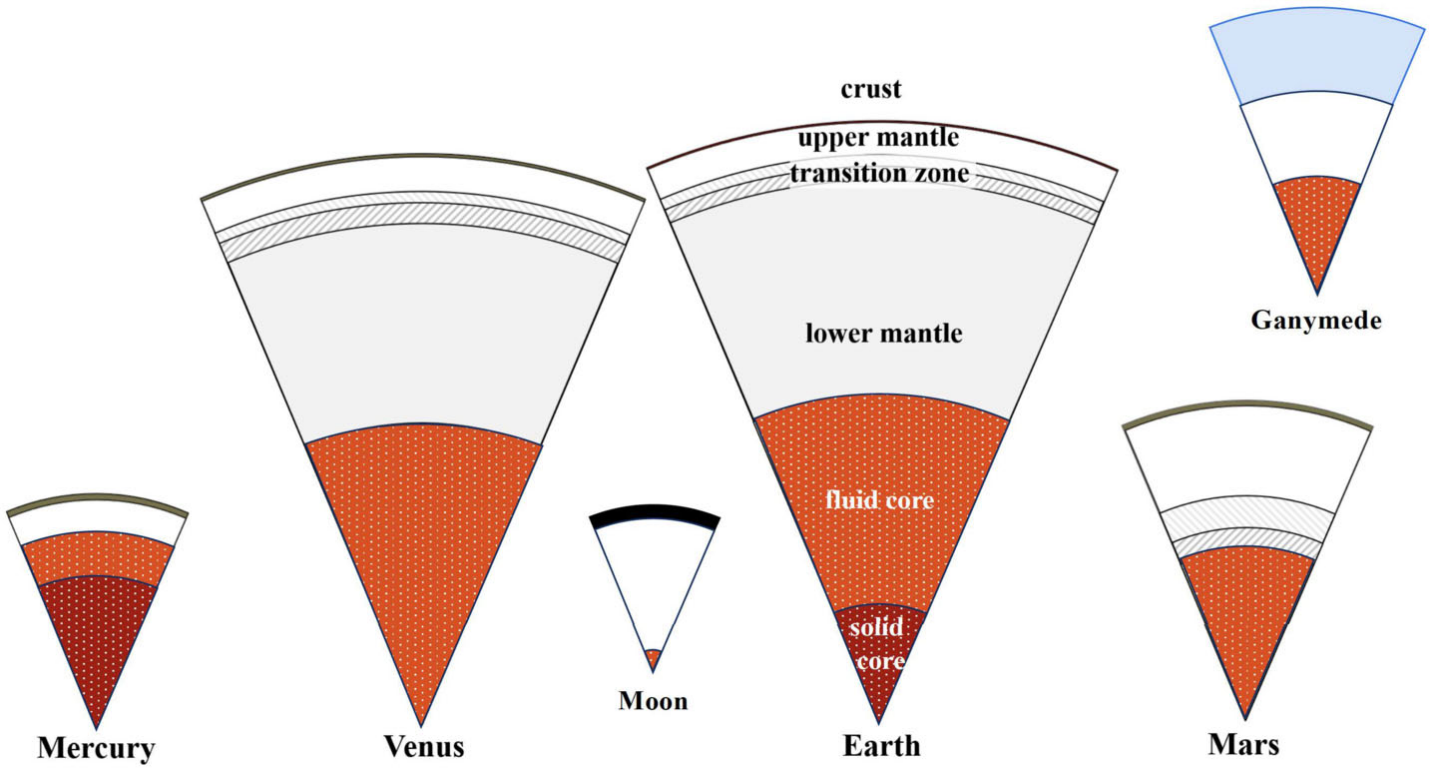
Interior structures of representative Solar System objects. The Earth, Venus, and Mars have iron-rich cores (red sections) with radii about half that of the planet. This is typical of other differentiated Solar System objects as well. For icy Solar System satellites, the ratio of 0.5 applies to the core radius relative to the satellite radius minus the ice shell thickness (blue). Mercury and the Moon have anomalous core-to-planetary radius ratios of approximately 0.8 and 0.2, respectively. Planetary cores are generally molten initially but may freeze as the planets cool. Earth and Mercury have solid inner cores (dark red). Venus, Mars, and Ganymede may have solid inner cores as well, although the absence of magnetic fields at Venus and Mars suggests that their cores may still be fully molten (Stevenson et al. 1983). The rocky mantles of Earth and Venus are compositionally layered with phase transitions (olivine-spinell and $\beta $ to $\gamma $ spinel) dividing the layers. These high-pressure phase transformations likely also occur in the lowermost Martian mantle. (Modified after Breuer and Spohn 2023)

**Fig. 3:**
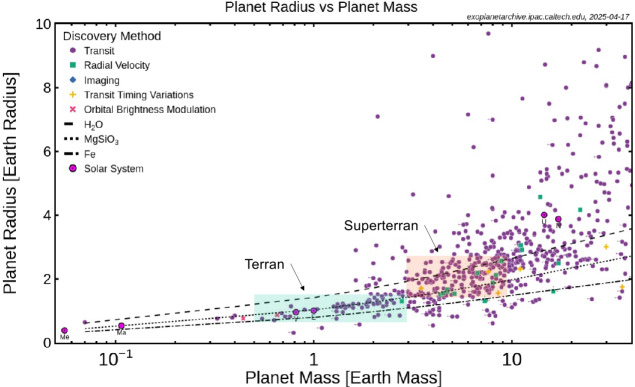
Radius versus mass for confirmed exoplanets as collected by Nasa’s Exoplanet Archive (https://exoplanetarchive.ipac.caltech.edu, 2025-04-17). The different observation methods are indicated. Also plotted are the masses and radii of Solar System planets (except Jupiter and Saturn), the radii of model planets composed of H_2_O, MgSiO_3_ and Fe, and the fields of terran and superterran planets. Note that generally errors are large, in particular in mass. Note that Venus, Earth, and Mars plot close to the MgSiO_3_ curve. See also Figs. 1 and 2 of Lineweaver and Chopra (2025)

Earth’s interior is divided into a rocky mantle (and crust) and an iron-rich core that freezes over time by growing a solid inner core (e.g., Hirose et al. 2013). The buoyancy released by freezing is widely agreed to drive a dynamo that generates Earth’s magnetic field (compare Sect. 2.1.3). The radius of the core is about half the radius of the planet, a characteristic shared by most rocky planets and satellites in the Solar System with the notable exceptions of Mercury and the Moon (compare Fig. 2). Even for the ice-rich satellites of the outer Solar System, the ratio of the iron–core radius to the satellite radius minus the thickness of the ice shell is about 0.5. The mass–radius statistics of exoplanets (Fig. 3) suggest that this ratio is more variable for rocky exoplanets. Earth’s solid inner core has grown over time to a radius of a little more than a third of the core radius. Geodetic data from the Messenger mission suggest that Mercury likely has a substantial solid inner core of 0.3 – 0.7 core radii (Genova et al. 2019). Solid Earth’s outermost layer, the crust, encompasses both the oceanic crust that underlies the oceans and the predominantly sub–aerial continental crust that constitutes the continents and continental shelves.

The interior structure of Earth, and to a lesser extent Mars and the Moon, is well understood thanks to seismological exploration (e.g., Dziewonski et al. 2015; Lognonné and Johnson 2015; Lognonné et al. 2019). However, the interior structure of other rocky worlds in the Solar System is much less constrained by mass, radius, gravity field, and tidal deformation (e.g., Sohl and Schubert 2015). This is even more true for rocky exoplanets (Baumeister et al. 2025). To infer the interior properties, in addition to mass and radius, the composition of the exoplanet’s central star is used, which is justified by the similarity of the compositions of the Solar System bodies with that of the Sun (e.g., Palme et al. 2014). The interior structure may indirectly influence (eu)habitability through its effect on tectonic styles, such as plate tectonics, and degassing and planetary magnetism, as discussed below.

##### Plate Tectonics and Continents

**Fig. 4:**
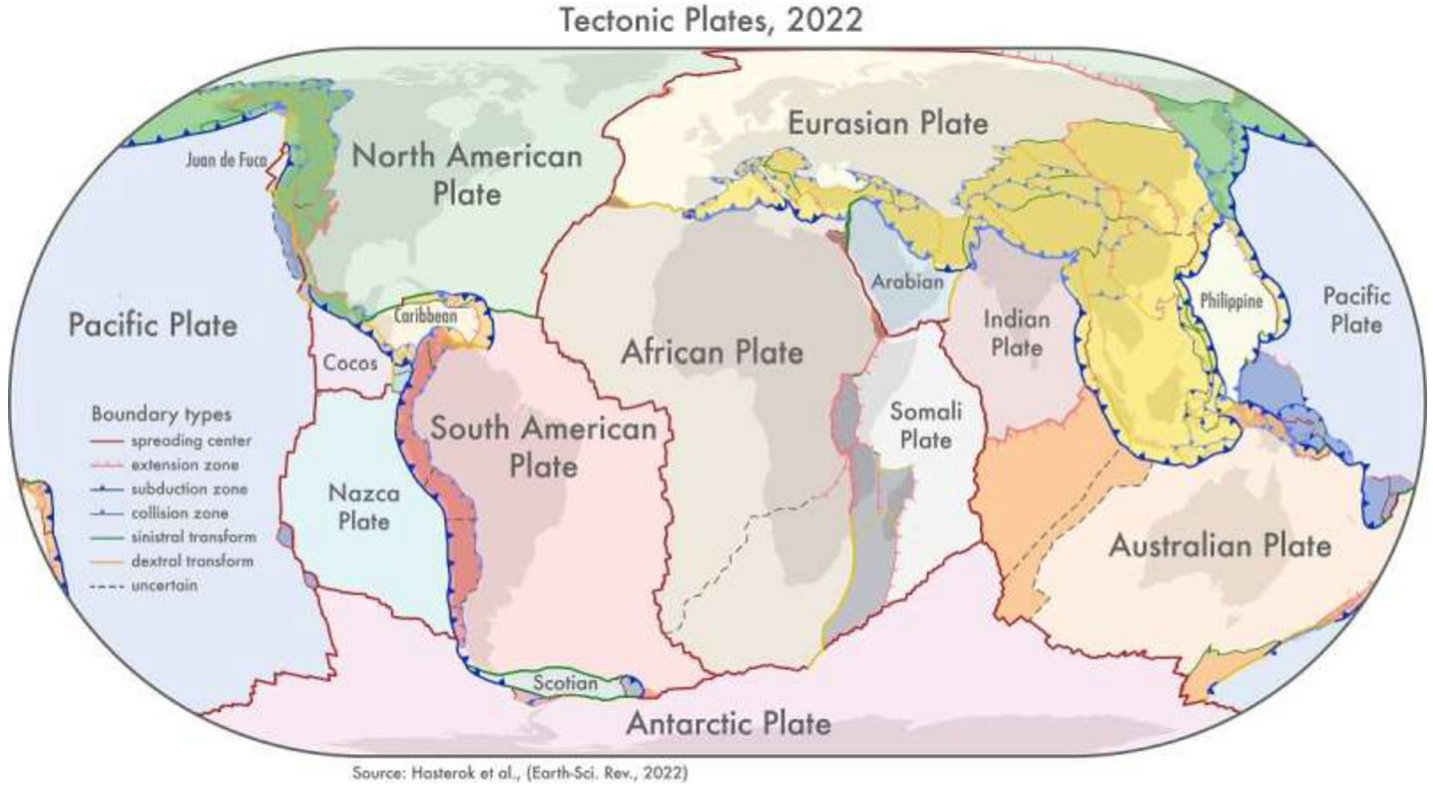
The tectonic plates of the Earth and their boundaries. There are seven major (African, Antarctic, Australian, Eurasian, North and South American, and Pacific) and several minor plates. Source: http://phys.org/news/2022-06-global-geological-provinces-tectonic-plates.html by D. Hasterok, University of Adelaide, based on Hasterok et al. (2022). Retrieved July 21, 2025

**Fig. 5:**
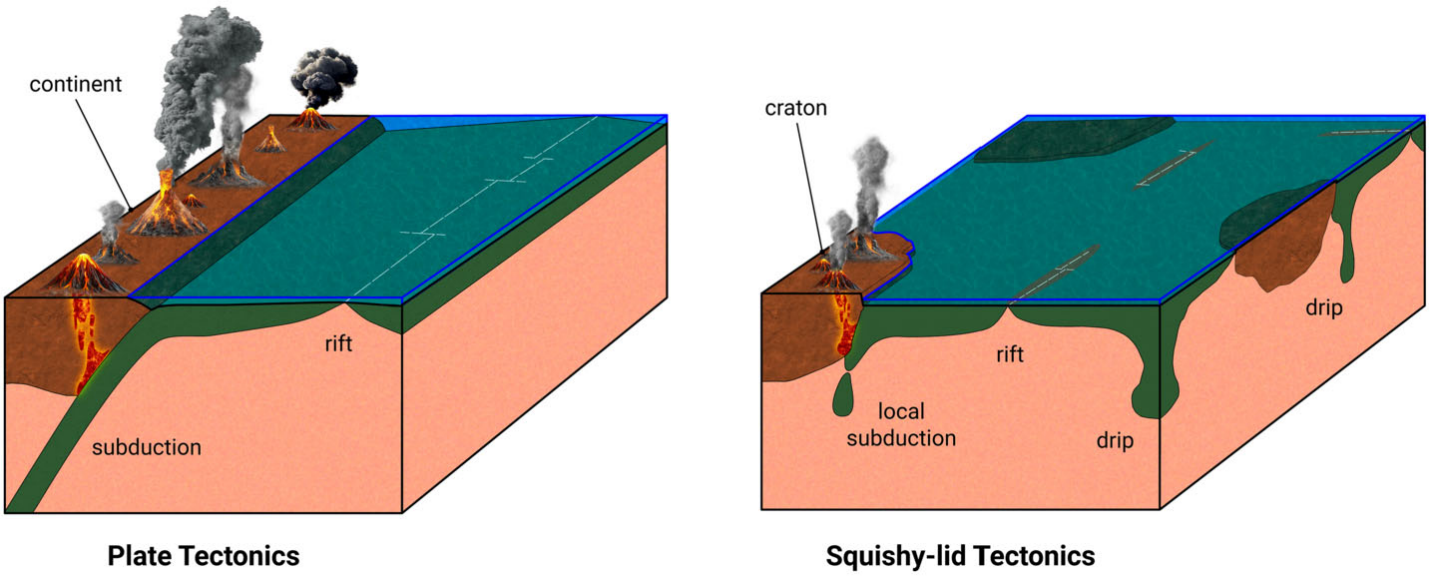
Schematic diagram illustrating modern plate tectonics and continental growth by subduction-related volcanism, as well as earlier squishy lid tectonics. Squishy lid tectonics may have predated plate tectonics during the early evolution of the Earth in the Archean when the lithosphere was warmer and more deformable. This, in turn, may have been predated by heat-pipe tectonics in the Hadean and possibly the early Archean. In this model, a stagnant lid is pierced by volcanic upwelling, and the mass balance is maintained by lithosphere delamination. Transitions between heat pipe, squishy lid, and plate tectonics were likely gradual. (modified after Cawood et al. 2022)

**Fig. 6:**
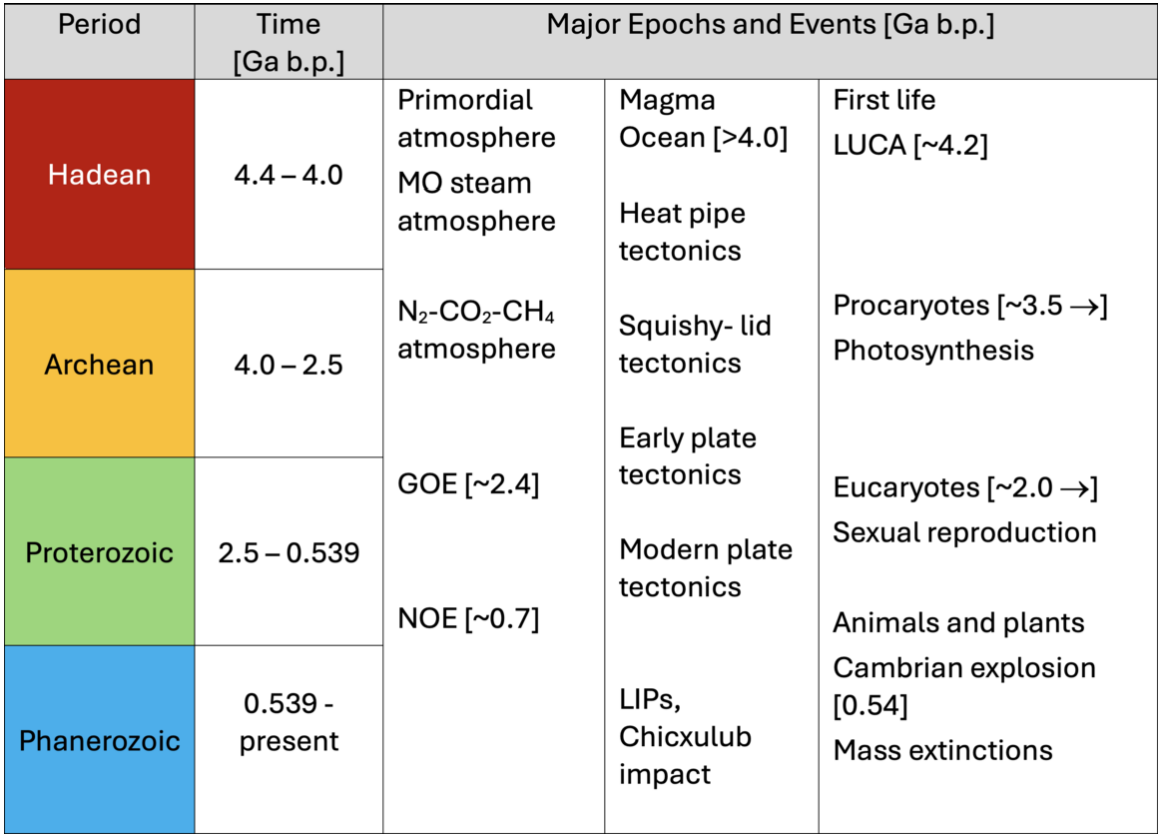
Simplified geological time table and major epochs and events used and discussed in the present paper. GOE denotes the great oxdation event and NEO the Neoproterozoic oxidation event

Heated by radiogenic elements and losing heat from accretion (the present–day ratio in the surface heat flow is about 1:2 (e.g., Jaupart et al. 2015)), Earth’s mantle undergoes solid state convection with flow rates of cm/year. This convection drives plate tectonics (see Lourenço et al. 2026, this topical collection, and e.g., Sleep 2015), a tectonic mode that appears to be unique at the geological present in the Solar System. Plate tectonics, wherein the cold and stiff upper layer of seven major rigid plates moving horizontally across Earth’s surface participates in mantle convection flow through plate subduction (compare Figs. 4 and 5), is widely agreed to be of significant importance to Earth’s biosphere (e.g., Langmuir and Broeker 2012; Cockell et al. 2016; Spencer 2022; Lingam and Balbi 2024; Stern 2016), with feedback from life. It can be argued that its characteristic feature, the rigid plates that make up the lithosphere, is not as important in this respect as the associated geochemical cycling between the interior and surface reservoirs. For this reason, it is not so important to know when modern plate tectonics exactly began, although estimates range from after the crystallization of the magma ocean (e.g., Korenaga 2021a) to some time in the Proterozoic less than 2.6 Ga ago (e.g., Cawood and Hawkesworth 2019), or as late as in the early Phanerozoic (e.g., Stern and Gerya 2024), 0.54 Ga ago (see Fig. 6 for a compilation of the geological time table as used in this article). What is more important is that earlier tectonic styles, such as squishy–lid tectonics (e.g., Lourenço et al. 2020, and compare Fig. 5), likely involved similar material cycles and rates of heat transfer. Taken together, these tectonic styles might be better termed “mobile–lid tectonics”, although “plate tectonics” as it is often used in planetary science, means just that. The tectonic cycle produces the Earth’s oceanic and continental crusts, transfers basic nutrients to the surface, and buffers the mass and composition of the oceans. It has been argued that Mars (e.g., Gregg 2015) and Venus (e.g., Harris and Bedard 2014; Rolf et al. 2022) exhibited some form of mobile–lid tectonics in their early evolutionary histories but currently show no evidence of large–scale horizontal lithospheric movement. Instead, Mars, Mercury and, possibly, Venus are usually cited as examples of stagnant–lid tectonics to describe a tectonic style without large-scale horizontal movement of lithospheric units and very limited cycling. It should be noted that an immobile lid covering a convective layer is the natural outcome of convection in a fluid with strongly temperature–dependent viscosity (e.g., Schubert et al. 2001).

Rock, being a multi-component chemical system, partially melts at temperatures above the solidus temperature, releasing buoyant melt that can rise to the surface where it crystallizes as basaltic rock to form the planetary crust. On Earth, because of the near-surface layers involved in convection, (hydrated) basaltic crust is subducted into the mantle, where it partially melts in the presence of water and sediments to form felsic crust that builds up into a thick layer of mostly sub–aerial continental crust. The latter is generally less dense than the basaltic crust, is comparatively stable, and rises about 2 km on average above the sea floor. Where the continental crust has been tectonically deformed (e.g., the Alps and the Himalayas), it can rise up to 10 km above the sea floor. The continents thus cause the Earth’s hypsometric curve to have two peaks and mostly rise above the ocean waters that naturally fill the basins provided by the basaltic crust, hence the term “oceanic crust”. In the Archean, formation of the oldest parts of the continents, the cratons, may have involved melting induced by mantle upwelling and the injection of mafic crust by volcanism. Zircon data suggest the formation of felsic rock as early as 4.1 Ga ago (e.g., Wilde et al. 2001; Mojzsis et al. 2001; Valley et al. 2002). Chowdhury et al. (2025) argue that the hypsometry evolved through the Archean during which the cratons became sub–aerial. However, it is important to acknowledge the paucity of rock records from the Archean eon, a consequence of continental erosion and crustal recycling.

The continental crust covers about 40% of the present Earth’s surface and may have been so for most of the geological past (e.g., Chowdhury et al. 2025). Since much of the low-lying continental margins are submerged, the part of the Earth’s surface above sea level is 29% of the total. The division of Earth’s surface into oceans and land has been important for terrestrial life since at least the Cambrian (e.g., Awramik and McNamara 2007; Simpson 2017; Lingam and Loeb 2021). Certainly the water in the oceans is important because life needs water, but most life, at least life as we know it, is directly or indirectly phototrophic – it lives off the sun’s radiation. The importance of land surfaces is illustrated by the large proportion of total biomass that is land-based life. Land-based biomass exceeds oceanic biomass by two orders of magnitude, even though the dead fraction of terrestrial biomass is 2/3 of the total (Bar-On et al. 2018). Furthermore, terrestrial biomass mostly consists of autotroph producers, whereas much of oceanic biomass (oceanic referring to the deep ocean) contains heterotroph consumers of terrestrial biomass. This extends the reach of phototrophy into the dark realms of the deep ocean, where some work shows that there may even be an obliquity dependence when we consider an exoplanet context (e.g. Lerner et al. 2025).

But it is not just the availability of sunlight that favors continental areas. Important basic nutrients such as phosphorus and nitrogen are provided by the weathering of continental rock (e.g., Glaser et al. 2020; Guimond et al. 2026) and are transported to the oceans by runoff and rivers. Seafloor weathering can also provide nutrients, but at much lower rates, as argued by Lingam and Loeb (2019a) and Glaser et al. (2020), due to higher flow velocities in rivers, increased pH in ocean waters, and the lack of erosion providing fresh, weatherable surfaces.

Having roughly equal proportions of land and oceans is an advantage for a planetary biosphere. As Lingam and Loeb (2019a) show, this maximizes the availability of both nutrients and water. However, it may not be a natural outcome of the long-term evolution of a plate tectonics planet. Simpson (2017) argued, based on Bayesian statistics, that the more likely outcome is an Earth entirely covered by oceans but cautioned that feedback mechanisms may cause a mostly land covered planet. Höning and Spohn (2016, 2023) argued that, indeed, feedback in continental growth and water cycling between the interior and the oceans would make a planet almost entirely covered by continental crust a significantly more likely outcome than an ocean covered planet or a planet with an Earth-like balanced ocean/land distribution. The latter outcome is the least likely, but it would maximize the length of continental arc subduction zones. Both land and ocean planets would be habitable, but the net primary productivity^7^ would be only about one percent of Earth’s, and the O_2_ productivity might even be insufficient for oxygen-based life.

The continental crust cycle of production and erosion is linked to the water cycle; both are part of plate tectonics geochemical cycles. Water is transported in the oceanic crust and its sedimentary cover to subduction zones where much of it is carried to depth within the subducting slab, stored in hydrous minerals and directly in pores. Some of this water is released at depths between 100 and 200 km, where important hydrous minerals such as serpentine break down and release water, contributing to the formation of new continental crust. The water that is subducted deeper is absorbed by the mantle (which is far from saturated with water), reducing the viscosity of the mantle and thus working against the effect of the decreasing temperature to increase viscosity and decrease the speed of mantle convection. The continental crust is eroded by surface weathering and subcrustal erosion, with some of the sediments subducted with the slab. At present and probably for the Phanerozoic, both the growth and erosion rates of the continental crust and the rates of water de- and regassing have been roughly in equilibrium. It should be noted that the present-day distribution of continental crust surface ages (Goodwin 1996) can be qualitatively explained by assuming that crust production is proportional to plate speed and that erosion is proportional to the continental surface area even if the continents formed early and have persisted since then (Korenaga 2021a; Guimond et al. 2026). Thermodynamically, an equilibrium would be favored where the length of subduction zones is minimized, as for a planet that is either almost entirely covered by oceans or by land. Instead, the Earth operates with the total length of the subduction zones almost maximized, close to an equilibrium which, as Höning et al. (2019) and Höning and Spohn (2023) have shown, is stable with respect to the water cycle but unstable with respect to the continental crust cycle. In the language of non-linear system dynamics, the Earth operates and evolves close to and along an unstable fix point of the system, maximizing the rate of entropy production.

It is widely accepted that mobile–lid tectonics effectively cools the interior by cycling cold lithosphere with the deep interior. Thereby, mobile lid tectonics effectively powers the core dynamo. The buoyancy released as the core cools and freezes is directly related to the rate at which the mantle cools the core as we will discuss further below in Sect. 2.1.3.

#### Atmosphere and Oceans

We give a brief overview of the history of Earth’s atmosphere (see Steinmeyer et al. 2026 in this collection for a more detailed discussion) and ocean to the extent possible given that there is less and less information available as one moves farther and farther back in time.

The formation of the Earth’s early atmosphere and ocean, which sets the stage for its early habitability, remain a topic of debate and intense discussion (see Salvador et al. 2023 for a recent review). The canonical view in recent decades has generally been that after the dispersal of an early nebular atmosphere (e.g. Mizuno et al. 1978; Hayashi et al. 1979) a post-accretion magma ocean atmosphere would form. This atmosphere would have consisted mostly of CO_2_ and/or N_2_ with large amounts of water vapor – typically termed a magma ocean steam atmosphere. Within ∼10^6^ years the surface and atmosphere would cool sufficiently for the steam atmosphere to condense out on the surface forming Earth’s first oceans (e.g. Hamano et al. 2013; Salvador et al. 2017). In the past few years the composition of the magma ocean atmosphere has come under scrutiny. Models show that these atmospheres can differ greatly depending on the redox state, C/H ratio, and water inventory of the mantle (e.g. Bower et al. 2022; Maurice et al. 2024; Nicholls et al. 2024). These magma ocean atmospheres influence both when and how Earth’s first oceans formed. They would also influence the composition of the post-magma ocean atmosphere, which could be dominated by either N_2_, CO_2_, CO, CH_4_, or H_2_O with minor contributions from any of those alongside H_2_ (Bower et al. 2022; Maurice et al. 2024). Here it is clear to see how constraints on Earth’s magma ocean atmosphere may play into exoplanet atmosphere observations in similar epochs, and perhaps vice versa.

If indeed the canonical picture is correct then a few interesting observations can be drawn from the data and associated models. Firstly, there is support for what has been termed a “Cool Early Earth” (Valley et al. 2002) with liquid water present on Earth’s Hadean surface supported by zircon measurements (e.g. Mojzsis et al. 2001; Wilde et al. 2001; Valley et al. 2002). Although exactly how much of the surface was covered with liquid water (as opposed to ice if the planet were relatively cold) cannot be well established from the available zircon data mentioned above (e.g. Korenaga 2021b). At this point we should also point out the effect of ocean salinity on climate and habitability (Olson et al. 2022; Langmuir and Broeker 2012). Salt in ocean water leads to climate warming (via a lowering of the freezing point of water) but too much salt has negative effects on most terrestrial biology. Yet, the estimates of salinity (Marty et al. 2018) and pH (Krissansen-Totton et al. 2018) in the Archean are quite poor (e.g. Catling and Zahnle 2020). Regardless, it is not difficult to see that, in the early years of the Hadean era, the Earth’s ocean was likely covering a fairly flat surface with limited or no exposed land. Different publications give varying results for the amount of exposed land through time (e.g., Korenaga et al. 2017; Cawood et al. 2022; Rey et al. 2024; Chowdhury et al. 2025), and this topic is covered in detail in another article in this collection (Guimond et al. 2026). In the short term (the next decades) this is an area where exoplanet observations could provide direct insights that we have yet to glean from Earth’s record, whereas for the most part it is Earth that provides insights into exoplanets. Hence, if we can make similar observations of Earth-like worlds in their early post-accretion phases (see Sect. 3) we may begin to get information on the early ocean/land ratios of worlds in their equivalent early to late Hadean phases.

The exact composition of Earth’s Hadean atmosphere remains largely unknown. However, there are better constraints for the Archean (e.g. Catling and Zahnle 2020, for a recent review). Assuming the mid-to-late Hadean atmosphere resembles the Archean atmosphere, one would expect an N_2_ dominated atmosphere with partial pressures of CO_2_ of up to 10 s of percent in a ∼ 1 bar atmosphere. These large CO_2_ partial pressures would be necessary to counteract the lower luminosity of the Sun at that time: ≲ 75% of modern (e.g. Gough 1981; Claire et al. 2012). The need for higher concentrations of greenhouse gases in the Hadean and Archean eras compared to the present day was originally referred to as the Faint Young Sun Paradox (Ringwood 1961; Sagan and Mullen 1972). A variety of other greenhouse gases were proposed, such as CH_4_ and NH_3_ (Sagan and Mullen 1972). However, Kuhn and Atreya (1979) showed that NH_3_ was unstable in the atmosphere of early Earth. Neither can one simply rely on CH_4_ because, once ratios of CH_4_/CO_2_ reach values of ∼0.1 or higher, hydrocarbon hazes form and cool the atmosphere (Haqq-Misra et al. 2008). Other authors have proposed that a small increase in CO_2_, along with fewer clouds and a resultant lower albedo, may have been sufficient (e.g. Rosing et al. 2010; Goldblatt et al. 2021), as opposed to higher CO_2_ partial pressures, which may not be supported by the geological record (e.g. Rosing et al. 2010). More recent work demonstrates that CO_2_ amounts in the Archean could have been as much as 2500 times higher than modern levels, while CH_4_ could have been as much as 10^4^ times higher (e.g. Catling and Zahnle 2020). We also have proxies that give us estimates of atmospheric density. A number of geochemical proxies for 3.0–3.5 Ga (e.g. Marty et al. 2013) give atmospheric pressures ranging from 0.5 – 1.1 bar with CO_2_ partial pressure values less than 0.7 bar. Work by Som et al. (2012) analyzing fossil raindrops from 2.7 Ga gave values of 0.51 – 1.1 bar depending on atmospheric composition. The fossil raindrop data in combination with later work (Som et al. 2016) utilizing the size of Archean lava vesicles gave a value of 0.23 ±0.23 bar (2$\sigma $) with an upper limit of ∼0.5 bar atmospheric pressure. If Earth’s atmospheric pressure indeed changed so dramatically through the Archean, it is important for exoplanet researchers to consider its impact on the greenhouse gases required to maintain a temperate climate (e.g. Charnay et al. 2017).

The Archean atmosphere was likely to be reducing, with significant amounts of methane, although limited by the possibility of haze formation as mentioned. Methanogensis dates back to more than 3.5 Ga ago (Wolfe and Fournier 2018), giving a methane source through much of the Archean where O_2_ level remained below 10^−6^ until ∼ 2.4 Ga (Catling and Zahnle 2020). With rising levels of O_2_ during the great oxidation event (Holland 2002; Lyons et al. 2014) (and possibly before, Kasting 2025), methane would have been destroyed along with its greenhouse effect and this is often cited as the reason for the “snowball” Earth near the start of the Proterozoic, which has been modeled with 3-D general circulation models (e.g. Feulner et al. 2023) and which could possibly be detected on exoplanets in the future (e.g Cowan et al. 2011; Herbort and Sereinig 2025).

The atmosphere of the Proterozoic is better understood, but there remain uncertainties especially with respect to O_2_ abundances with estimates ranging from <1% to >10% present atmospheric level (e.g. Lyons et al. 2014, 2021; Reinhard et al. 2016; Mukherjee et al. 2025). There do not appear any recorded large excursions in surface pressure, with N_2_ remaining the main component of the atmosphere. O_2_ is not a radiatively active gas (like N_2_), and hence is not a greenhouse gas like CO_2_. However, at larger concentrations it can contribute to line broadening of greenhouse gases (as $\mathrm{N_{2}}$ does) and hence increase their effectiveness (e.g. Goldblatt et al. 2009). This is taken into account in radiative transfer codes in 3-D general circulation models. Most models show that the land-to-ocean ratio has stabilized in the Proterozoic and is roughly similar to that of today as shown in e.g., Cawood et al. (2022) and in Guimond et al. (2026) in this topical collection). Perhaps the most interesting aspect of the Proterozoic is the rise of more complex eukaryote lifeforms instead of simple single–celled prokaryote organisms like stromatolites or cyanobacteria. While the latter had substantial impacts on the atmosphere (e.g., by the production of methane, a potent greenhouse gas), the former had even more with the continuing rise of photosynthesis and changes in ocean and land biogeochemistry. These latter changes would have, to first order, influenced surface composition, albedo, and cloud cover that could have direct exoplanet observational consequences (e.g. Cowan et al. 2009). Additionally, two other large scale events with dramatic climate impacts can be quantified in this epoch: the supercontinent cycle (e.g. Jellinek et al. 2020) and snowball Earth (e.g., Hoffman and Li 2009). An exoplanet in an Earth-like snowball state will be observationally distinct from that of a more temperate world (e.g. Cowan et al. 2011). Snowball states tend to be characterized by extremely high surface albedos, dryer atmospheres and an associated decrease in cloud cover (e.g. Hyde et al. 2000). The supercontinent cycle has also been associated with large igneous provinces (LIPs), as discussed by e.g., Ernst et al. (2013). These topics will be discussed in more detail in the paragraphs on the Phanerozoic below.

Finally, we reach Earth’s most recent geological epoch, the Phanerozoic (∼ 540 Ma to present), which includes the Ordovician and the more well-known Cambrian explosion which eventually gave rise to dinosaurs, reptiles, mammals and, of course, humans with their dramatic influence on Earth’s present climate. When non-specialists think about Earth, this is likely the only epoch they are aware of, and hence it is often considered the canonical Earth. Yet, as we’ve seen in this section, it is only a small slice of Earth’s entire history. Oxygen level estimates for the first half of the Phanerozoic tend to be higher than that of the Proterozoic, but in the latter half they are consistent with the present level. The Phanerozoic is the period in Earth’s history about which we know the most. For example, we know that LIPs have been responsible for most mass extinction events in the Phanerozoic (e.g. Wignall 2001) with dramatic environmental consequences (e.g. Black et al. 2024) that include the release of gases toxic to life, large increases in greenhouse gases, and associated warming which could melt most surface ice affecting a planet’s surface albedo.

In general, it is widely believed that impactors have played a major role in mass extinction events throughout history. However, let us look at the most famous mass extinction example at the End-Cretaceous (the Cretaceous–Paleogene (K–Pg) extinction event, also known as the Cretaceous-Tertiary (K–T)) ∼ 66 Ma. This is probably because of the outsize role of the Chicxulub impactor in the demise of the dinosaurs due to its environmental consequences (e.g. Morgan et al. 2022).^8^ However, at the time of the Chicxulub impactor the Deccan Traps were being formed via an extremely large LIP. The dating of the impactor and the LIP are close in time (e.g. Sprain et al. 2019; Schoene et al. 2019) and hence the on-going LIP may have already been weakening many species before the impactor delivered the *coup de grâce*. Yet, there remain some doubts as to whether an impactor played the major role in the End-Cretaceous mass extinction event (e.g. Bond et al. 2014). It has long been assumed that Jupiter may play a “protective” role for Earth by preventing asteroids and comets from entering its orbit (e.g. Wetherill 1994), although recent work has put this into question (e.g. Horner and Jones 2008, 2009; Horner et al. 2010).

It has also been estimated that temporally overlapping large LIPs could possibly drive an Earth-like world toward that of a runaway greenhouse as on modern Venus (Way et al. 2022). Hence, while most astronomers are focused on finding temperate Earth-like worlds in the habitable zones of nearby stellar systems, it could be equally likely that we find more Venus-like worlds for reasons scarcely explored. Earth’s climate over the past billion years has oscillated between quite warm climates like that of the Cretaceous and during LIP outgassing, to snowball states as documented in the Proterozoic above. In fact LIPs may even hasten the end of snowball states (e.g. Lan et al. 2022).

Another consequence of a focus on the Phanerozoic is the issue of the carbonate-silicate cycle and the role of modern subductive plate tectonics and volcanism originally postulated by Walker et al. (1981). Steinmeyer et al. (2026) in this topical collection discuss this in detail. Here we point out the importance of the carbonate–silicate cycle for weathering, the rate of which depends on temperature and CO_2_ partial pressure. Nutrient cycles of nitrogen (e.g. Förster et al. 2019; Berner 2006; Stüeken et al. 2016), phosphorus (e.g. Alcott et al. 2022, and references therein), and other cycles (e.g. Alcott et al. 2019), depend on weathering through carbonic acid. Walker’s original paper speculated that this cycle could have been responsible for stabilizing Earth’s climate over the majority of its history, and played a role in addressing the Faint Young Sun Paradox mentioned above. While it is uncontroversial to say that Earth has had a form of volatile cycling throughout much of its history, the idea that modern volatile cycling is the same as that of the late Hadean or early Archean is probably misplaced. First, the amount of subaerial land at that time is the subject of intense debate, leaning towards there being less than today. This would impact the land–based carbonate-silicate cycle. One could only rely on seafloor weathering (e.g. Chambers 2020) in the late Hadean to early Archean. Recent work argues that seafloor weathering is not as effective as land weathering (Lee et al. 2019; Glaser et al. 2020; Ouyang et al. 2025). In addition, it is questionable that Earth’s tectonic state has always remained the same as today (e.g. Cawood et al. 2022, Fig. 17). For example, the Hadean could have been in a “heat pipe” state (Moore and Webb 2013). It has even been speculated by Moore et al. (2017) that “Terrestrial exoplanets appreciably larger than Earth may remain in heat-pipe mode for much of the lifespan of a Sun-like star”, but this is controversial (e.g., Balmer and Noack 2021). It has been proposed that a plutonic squishy lid tectonic mode (Lourenço et al. 2018, 2020) was the dominant tectonic state from the late Hadean to the early Archean and that it may resemble the conditions on modern Venus (Rolf et al. 2022). The volatile cycle that occurs in such a tectonic state has scarcely been investigated although e.g., Foley and Smye (2018) argue that a carbonate-silicate cycle should be possible even with stagnant lid tectonics. In the end, we should be cautious in assuming that all Earth-sized terrestrial worlds have volatile cycling like that of modern Earth. It could be that many or even most are in a Venus-like plutonic squishy lid, a Mars-like stagnant lid, or even a Io-like heat-pipe mode with consequences on their ability to affect any long-term volatile cycling, even if shorter-term means have been proposed (e.g. Höning et al. 2021).

**Fig. 7:**
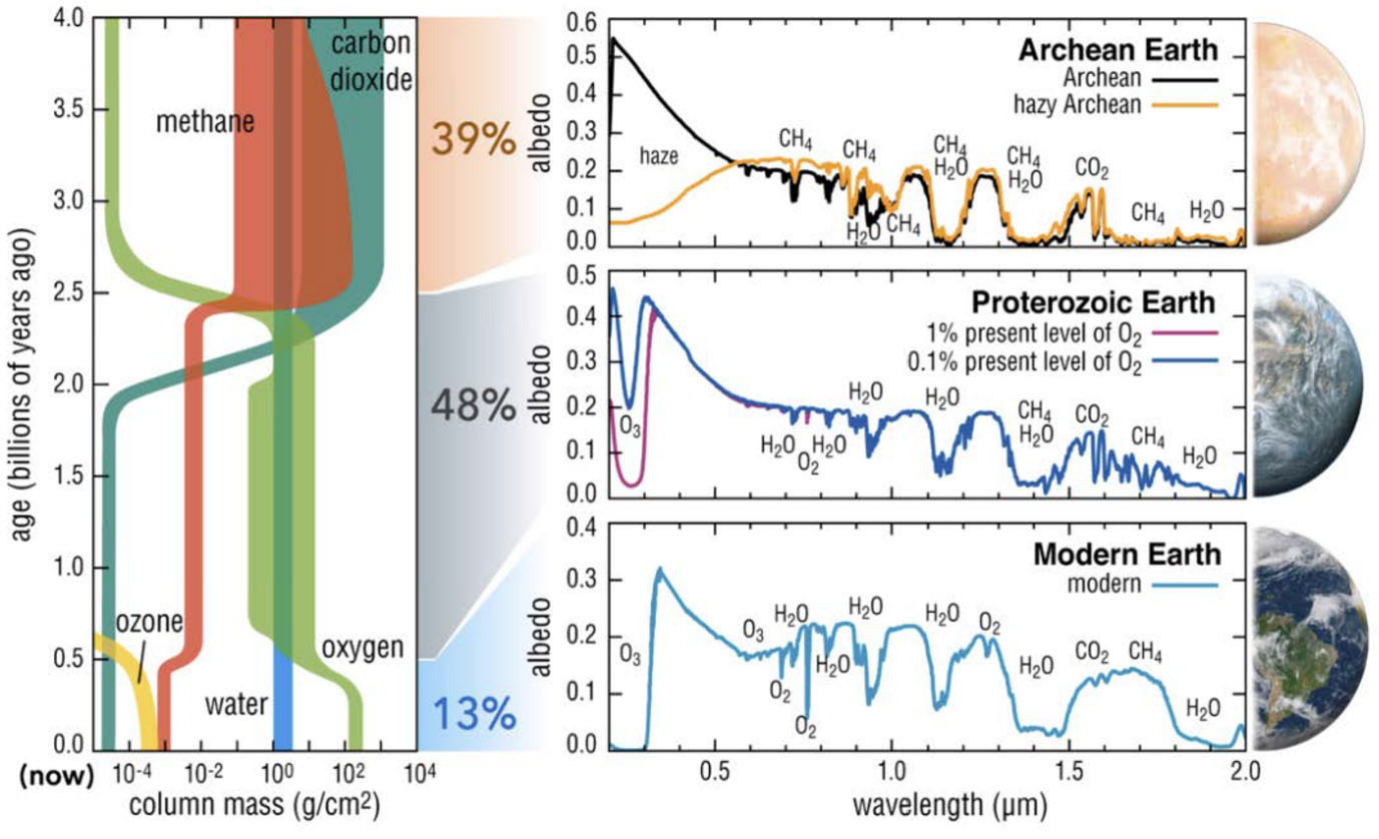
Earth’s planetary spectra through time as seen with the LUVOIR concept mission (LUVOIR Team 2019). Credit: G. Arney, S. Domagal-Goldman, T. B. Griswold (NASA GSFC)

These three main epochs in Earth’s history can be broadly demonstrated in simulated transmission spectra for the LUVOIR concept study (LUVOIR Team 2019) as shown in Fig. 7. For example, comparing the Archean and Proterozoic, the Archean does not show the most commonly assumed biosignature gases O_2_ and O_3_, since the Archean had so little O_3_ which is produced from O_2_. Comparing the Archean to the Proterozoic and modern Earth we see that CH_4_ is unsurprisingly more prevalent. When we compare the Proterozoic to the present day, we see that the higher abundance of O_2_ in the atmosphere today results in a much larger O_3_ absorption feature. This is one of the reasons why the LUVOIR mission concept was designed to be sensitive to such short wavelengths and is expected to be adopted for the Habitable Worlds Observatory (Clery 2023).

#### Magnetism

The importance of the Earth’s magnetic field in sustaining the biosphere is a debated topic. Certainly, evolved life has learned to use the field for navigation and other purposes. However, it is unclear whether a magnetic field is an essential element of (eu)habitability. Some argue that a sufficiently strong magnetic field can protect the atmosphere from solar (stellar) wind erosion and shield the biosphere from harmful cosmic rays (as discussed in Kubyshkina et al. 2026 in this topical collection), making it an essential requirement for habitability (e.g., Lammer et al. 2009; McIntyre et al. 2019). However, critical debates about this paradigm have emerged in recent years. These debates are based on spacecraft observations showing similar atmospheric loss rates on Solar System planets despite their differing magnetic field properties and on model calculations (e.g., Blackman and Tarduno 2018; Gunell et al. 2018; Ramstad and Barabash 2021; Way et al. 2023). The role of a magnetic field in protecting a planet’s surface from harmful radiation has also been questioned (e.g., Lingam and Loeb 2019b), while the importance of a thick atmosphere has been emphasized (Grießmeier et al. 2016).

Scaling laws have been proposed for predicting magnetic field strength based on observable planetary parameters. A critical review of the scaling laws and a comparison with numerical dynamo calculations is provided in Christensen (2010). Accordingly, the most convincing scaling law for the magnetic field strength was found to be independent of the planetary rotation rate but dependent on the energy flux in the dynamo region available to balance the Ohmic dissipation:

1 $$ B \sim R_{c}^{2/3}q_{c}^{1/3}, $$

where $B$ is the magnetic induction, $R_{c}$ is the core radius (for a terrestrial planet), and $q_{c}$ is the buoyancy flux in the core. For a thermally driven dynamo, $q_{c}$ is proportional to the heat flow from the core. For a dynamo driven by chemical convection, $q_{c}$ is proportional to the rate of growth of the inner core. Depending on how the dynamo is driven, the efficiency of converting energy into magnetic field energy, and whether the field has a strong dipole component or is largely multipolar, the constant of proportionality in the above equation takes values between 0 and 1 (see Christensen et al. 2009 and Christensen 2010, for discussion). In the latter case, the field strength is only about half that of a strong dipole field. A thermally driven dynamo would have a field strength that is only a fraction of that of a chemically driven dynamo.

The topology of the field has been found to depend on the strength of the inertial forces relative to the Coriolis force in dynamo experiments and thus on the rotation rate. This is particularly relevant for tidally locked exoplanets, where the rotation rate is equal to the orbital frequency, which is much smaller than Earth’s rotation rate, for example. However, these planets may still have substantial magnetic fields, albeit multipolar, that could protect them from radiation and stellar winds if their core and interior evolution is similar to Earth’s.

Both terms on the right-hand side of Eq. (1) depend on the mass of the planet under consideration. Therefore, it is conceivable that there is a lower mass limit for rocky bodies to sustain a self-generated magnetic field. However, Bryson et al. (2015) use paleomagnetic data from pallasites to suggest that asteroids with a radius of 400 km could have thermally and chemically driven dynamos. Furthermore, it has been demonstrated that asteroids with radii of tens of kilometers could possess differentiated iron-rich cores and support early dynamos if they accreted rapidly enough relative to the 0.7 Ma half-life of ^26^Al (Neumann et al. 2013). At the other extreme, while some suggest that super-Earths should cool efficiently and have magnetic fields (see e.g., Balmer and Noack 2021, for a discussion), others have argued that the pressure effect on mantle rock rheology may prevent deep mantle convection, efficient core cooling, and core dynamo action (e.g., Stamenković et al. 2012). Notably, Mars once had an intrinsic magnetic field, as evidenced by remnant crustal magnetism (e.g., Acuña et al. 1999). Mercury and Ganymede both have intrinsic magnetic fields (Anderson et al. 2010; Kivelson et al. 1996). But Venus, which is a similar size to Earth, has no magnetic field. Some believe that any remnant crustal magnetization could be detected if Venus once had a magnetic field (O’Rourke et al. 2019).

#### Life

Despite all efforts to date, life beyond Earth has not yet been detected. In the Solar System, primitive carbon– and water–based life may be detectable at certain depths in Martian soil, where the ambient temperature would permit the presence of liquid water. Similar favorable conditions may exist in the subsurface oceans of Europa and Enceladus. Exotic life forms that utilize solvents other than water may be discernible on Titan’s surface or within the clouds of Venus (e.g., Schulze-Makuch and Irwin 2018). These worlds offer urable and habitable niches in which life may have originated and evolved. Considering the Earth, one may inquire how urable and (eu)habitable conditions may have *determined* the origin of life and its evolution to technological intelligence, and to what extent life was subject to *contingency*. In other words, would life on an exoplanet with similar properties invariably evolve in an analogous manner to that of Earth?

**Fig. 8:**
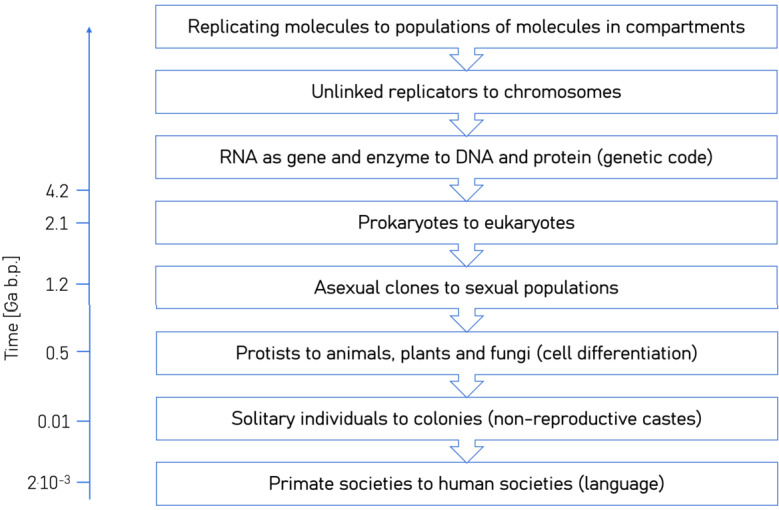
The major transitions in the evolution of life on Earth up to human societies, as compiled by Szathmáry and Smith (1995). The first three transitions occurred prior to LUCA. Estimates of when the steps were taken are given

Following Smith and Szathmáry (1995), Szathmáry (2015), Knoll and Bambach (2000), and Henrich (2020), we briefly outline the major stages of the evolution of life on Earth (compare Fig. 8) and discuss *contingency* and *convergence*^9^ in evolution. It is widely agreed that life originated in the Archean, possibly the early Archean, and consisted mostly of prokaryotes – archaea and bacteria. The last universal common ancestor (LUCA), the fundamental node in the tree of life, from which the domains of archaea and bacteria diverged, has recently been dated to $4.2^{+1.3}_{-1.1}$ Ga b.p. (Moody et al. 2024). Accordingly, LUCA was an anaerobic acetogen that lived in an established ecological system that it may have supported. During the Archean era, cyanobacteria or their predecessors invented (oxygenic) photosynthesis and simple forms of multicellularity originated in mats and filaments. The Proterozoic era saw the origin of eukaryotes followed by sexual reproduction and the origin of complex multicellularity (animals and plants) during the Neoproterozoic era (1 – 0.539 billion years ago) or the early Phanerozoic. According to the fossil record, life then spread to continental land areas, the Cambrian radiation, followed by the advent of intelligence and eusociality in hominids (living in multigenerational groups with a division of labor) about 2 million years ago. Finally, language, cumulative culture and technological intelligence emerged in humans through culture–gene–environment coevolution (e.g., Durham 1991).

Following the highly influential work of S. J. Gould (Gould 1989), there has been much debate on whether evolution in general is primarily contingent or convergent. Gould advocated for the primacy of contingency. Others have emphasized the deterministic nature of Darwinian evolution, converging toward the best adaptive solution to an evolutionary challenge (e.g., Morris 2003). Blount et al. (2018) reviewed the debate, including the proper meaning of contingency and cited Desjardins (2011), who clarified that contingency is intrinsic to path-dependent systems. In such systems, multiple possible paths from an initial state exist that lead to multiple possible outcomes with “probabilistic causal dependencies” linking the two. Blount et al. (2018) further reviewed substantial laboratory research and paleontological evidence on the subject, finding evolution to be both contingent and convergent. Close lineages tend toward similar adaptations, while distant lineages find distinct solutions. This resembles the dependence of deterministic chaotic systems on initial conditions, as described by e.g., Lorenz (1995).

The question of whether evolution is contingent or convergent is particularly relevant to the astrobiology of exoplanets because convergence could imply that advanced life is widespread throughout the universe (e.g., De Duve 2011; Morris 2011). However, Blount et al. (2018) have observed that the island of New Zealand lacks native terrestrial mammals, thus arguing against convergence. If contingency were dominant, it would raise doubts about the widespread existence of life, since major evolutionary transitions have only occurred once in geological history (e.g., Lingam and Loeb 2021). Therefore, exploring exoplanets for signs of life is of great importance – the “next best thing to rewinding the tape of evolution”, to quote Powell (2020). It is interesting to note that Powell (2020) acknowledges contingency in evolution, yet argues for the convergence of the evolution toward complex minds if complex bodies were common. Assuming convergence in the evolution toward complex minds led Lingam and Loeb (2021) to suggest that technosignatures in interstellar space may be a more abundant signs of life than biochemical signatures on planets and moons.

However, recent anthropological research has cast doubt on the idea of determinism in human cultural evolution (e.g., Henrich 2020; Henrich and Muthukrishna 2021). While geographic and environmental factors may suggest determinism (e.g., Diamond 1997), other contingent cultural factors may date back centuries and have had far-reaching consequences. One such transformation is the shift from kin-based societies to more individualistic yet ultrasocial societies. Where this transformation occurred, it was followed by urbanization, the formation of premodern states, and widespread literacy. This was followed by the further specialization of labor, analytical and scientific reasoning, continuous innovation, the Enlightenment and the Industrial Revolution (Henrich 2020; Henrich and Muthukrishna 2021).

Even if determinism were inherent to biological and cultural evolution, the conditions of (rocky) planets may offer enough contingency to alter the evolutionary path. Chopra and Lineweaver (2016) and Lineweaver and Chopra (2025) even argue for a “Gaian bottleneck” that a biosphere must pass through very early on to avoid extinction due to runaway greenhouse cooling or heating before it can stabilize the climate. Earth may have simply been fortunate enough to pass the bottleneck. Moreover, consider the snowball Earth glaciations during the Proterozoic and the five major mass extinction events in the Phanerozoic. Various causes of mass extinction events are discussed (e.g., Bond and Grasby 2017), including LIPs and major impact events such as the Chicxulub event (Alvarez et al. 1980). These events caused major climate and ocean chemistry perturbations and consequently mass extinctions, as discussed above in Sect. 2.1.2.

On a less dramatic scale, some argue that continental drift drove the diversification of lineages and the current distribution of biodiversity (Pellissier et al. 2018; Gerya 2022). There is consensus regarding the feedback between the evolution of the biosphere and the planetosphere as they co-evolve (e.g., Spencer 2022; Stern and Gerya 2024). Perhaps the most prominent example of this is photosynthesis and the subsequent oxygenation of the surface and crust, which caused its own mass extinction. Additionally, the increased weathering rate due to the land-based biosphere (e.g., Ouyang et al. 2025) in the Phanerozoic may have significantly impacted the growth of continents and modern plate tectonics as argued in modeling studies of continental growth by Höning and Spohn (2016, 2023). In essence, even if biological evolution were entirely deterministic – which it is now considered false (e.g., Blount et al. 2018) – randomly timed environmental events in the planetosphere will cause contingency. These events are caused by deterministic chaos in nonlinear interior, Solar System, and galactic processes. Would we be writing this article if the Chicxulub event (or the Deccan Trap LIP) had not wiped out the dinosaurs?

##### Implications for the Detection of Extraterrestrial Life

Contingency in evolution, particularly cultural evolution, introduces an additional challenging factor that is nearly impossible to estimate when considering the likelihood of detecting intelligent life beyond Earth (see also Basalla (2006) for a critical discussion, in particular, on the perspective of communicating with extraterrestrial lifeforms). It is estimated that there are about $10^{11}$ stars in the galaxy. According to recent work by Quanz et al. (2022), Lammer et al. (2024) and Scherf et al. (2024), fewer than a third of these may have rocky planets in their habitable zones. According to Scherf et al. (2024), approximately 0.5 to 2% of stars can host a planet with an Earth-like atmosphere dominated by $\mathrm{N_{2}}$ and $\mathrm{O_{2}}$ with 1 to 10% $\mathrm{CO_{2}}$. These authors estimate the probability of these planets actually having such an atmosphere to be about $10^{-4}$. Accordingly, there should be approximately $10^{5}$ planets in the galaxy that could be considered habitable given the aforementioned atmospheric composition constraint. However, given other constraints, this number could be considerably smaller. It is difficult to say how likely plate tectonics will be, for example. The probability of a favorable land/water distribution was estimated by Höning and Spohn (2023) to be a few percent. Thus, the chances for an Earth-like biosphere may be slim, only 1 in $10^{8}$! This estimate does not consider the likelihood that such a biosphere would evolve technological intelligence.

Recognizing that Earth may be rare and that the likelihood of finding a second planet like Earth may be very small does not mean that searching for extraterrestrial planets and life is not worthwhile. Rather, it suggests that we should expand our horizons and look for a greater variety of planets, life forms, and biospheres (e.g., Schulze-Makuch and Irwin 2018).

## Observability of Phenomena at Interstellar Distances

The previous sections highlight the variety of potential phenomena on terrestrial exoplanets, and how “2-D” and “3-D” processes may profoundly impact a planet’s long-term habitability. However, interstellar distances limit us to one-dimensional characterization of exoplanets for the foreseeable future, as higher-order characterization requires capabilities far beyond current techniques and technologies (e.g., Turyshev 2025). Using today’s techniques, we can study the upper atmosphere annuli of terrestrial exoplanets with transit spectroscopy and their total thermal emission with secondary eclipses and phase curves. In the coming decades, unresolved direct spectroscopy of the entire planetary disk will enable us to investigate the deep atmospheres, and potentially the surfaces, of terrestrial exoplanets. The challenge will be to find signs of complex planetary processes within these datasets, which lack spatial resolution across planetary surfaces. The opportunity lies in the large and varied set of exoplanets with parameters and conditions beyond those in the Solar System. In this section, we explore how the processes discussed in this article and in this topical collection might be detectable on terrestrial exoplanets in the coming decades (see also Lagage et al. 2026 in this topical collection).

### Atmospheric Composition

Of all the characteristics that could be investigated in detail for terrestrial exoplanets in the not-too-distant future, atmospheric composition is the most promising. Current facilities can probe exoplanet atmospheres using transit spectroscopy. However, this technique is most effective when the planet-to-star radius ratio is large (i.e., large planets around small stars) and is most sensitive to the upper layers, which may not represent the bulk of the planet’s atmosphere. Furthermore, when probing the very small signals associated with terrestrial planets and their atmospheres, spectral contamination caused by the inhomogeneous surfaces of stars (i.e., starspots) introduces a significant source of systematic error that is difficult to overcome (e.g., Rackham et al. 2023).

Secondary eclipse observations and phase curves have successfully measured thermal emission from small exoplanets around low–mass stars (e.g., Kreidberg et al. 2019). By comparing the measured nightside surface temperature to the dayside temperature, these observations can reveal whether a planet has an atmosphere capable of large–scale heat transport. The rocky planets in the TRAPPIST-1 system that have been studied with the James Webb Space Telescope (JWST), TRAPPIST-1b, -1c, and -1d appear to lack such an atmosphere (Greene et al. 2023; Zieba et al. 2023; Piaulet-Ghorayeb et al. 2025). The upcoming Ariel mission will perform transmission and eclipse spectroscopy of approximately 1000 warm and hot planets ranging in size from super-Earths to gas giants.^10^ Again, the lower–mass super–Earth–sized planets in the Ariel target list preferentially orbit low–mass stars (M dwarfs); however, it is unknown whether these planets can be habitable. Even if some or all of the super–Earth–sized exoplanets are terrestrial, questions remain about whether rocky planets in the liquid water habitable zones of M dwarf stars can retain significant atmospheres over geologic timescales (e.g., Van Looveren et al. 2025).

Probing the atmospheres of terrestrial exoplanets around Sun-like FGK stars requires shifting to observations that can more effectively distinguish small planetary signals from starlight contamination. One approach currently used for giant planets leverages the spectral difference between stellar and planetary atmospheres; most stars do not exhibit molecular absorption features. Cross-correlating a high-dispersion molecular model with a star and exoplanet spectrum taken at high spectral resolution has achieved detections of molecules despite large amounts of stellar contamination (e.g., Landman et al. 2024). This technique can reveal a planet’s spin rate and the relative abundance of its atmospheric constituents. However, obtaining absolute molecular abundances is challenging. It is uncertain whether this technique can be used to study the atmospheres of terrestrial exoplanets with the James Webb Space Telescope (JWST) or the future Extremely Large Telescope^11^ (ELT) remains uncertain, though it is likely to be more effective for planets around low–mass host stars.

**Fig. 9:**
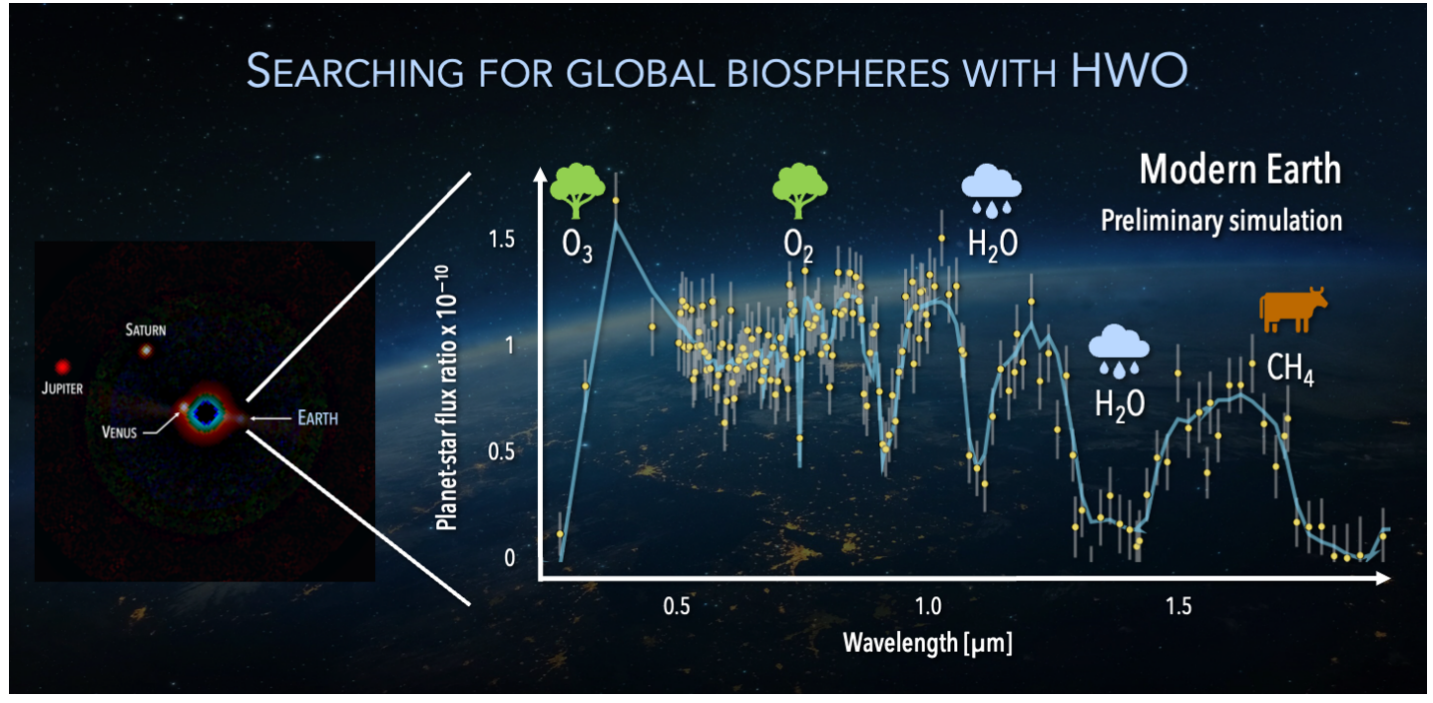
Preliminary simulated Habitable Worlds Observatory (HWO) high-contrast spectrum of a modern Earth-twin orbiting a nearby Sun-like star. This spectrum is likely of sufficient quality to measure abundances of key indicators of habitability (H_2_O) and atmospheric biosignatures (O_3_, O_2_, and potentially CH_4_ for earlier phases of the inhabited Earth). The total time to acquire this spectrum, in multiple observations, varies dramatically depending on the distance of the system from the Sun. Credit: J. Lustig-Yeager (JHU-APL), T. Robinson (U of Arizona), & G. Arney (NASA GSFC)

High-contrast direct spectroscopy, in which the bright host starlight is dramatically suppressed, is likely the best - perhaps the only - way to study the atmospheres of temperate, terrestrial exoplanets around Sun-like stars in the near future. Achieving the extremely high contrast required for such observations (planet-to-star flux ratios of less than $10^{-10}$ at visible wavelengths) demands a space-based telescope because the Earth’s atmosphere will likely limit the achievable contrast, even when using advanced coronagraphs paired with extreme adaptive optics systems on a future Extremely Large Telescope (ELT). The key goal of NASA’s Habitable Worlds Observatory (HWO) mission concept^12^ is obtaining direct spectra of potentially habitable exoplanets around FGK stars. Figure 9 shows a *preliminary* simulated direct spectrum of the modern Earth around a Sun-twin star as might be obtained with HWO.

### Atmospheric and Surface Biosignatures

The simulated direct spectrum of the Earth as an exoplanet in Fig. 9 helps us to understand what phenomena we will and will not be able to study in the coming decades. Molecular absorption features of water vapor (H_2_O), ozone (O_3_), and molecular oxygen (O_2_) are readily visible. Methane (CH_4_) is very difficult to detect at modern Earth concentrations, but could be more detectable at earlier phases in Earth’s evolution (the Archean eon; see Fig. 7). Ozone, molecular oxygen, and methane are all atmospheric biosignatures – gases produced by life at different concentrations over Earth’s inhabited history. Thus, signs of a global biosphere are potentially detectable on Earth-like exoplanets around Sun-like stars in the coming decades.

We emphasize, however, that no molecule is automatically a biosignature in every planetary and stellar context, as demonstrated by the healthy body of literature on potential biosignature false positives. The key to robust atmospheric biosignature identification is to have enough information to reliably model the source and sink rates of various gases in the absence of biology and look for anomalously high molecular abundances that point to an additional source. This will require accurate knowledge of the input stellar spectrum across a wide range of wavelengths, among other planet characteristics. Meadows et al. (2022) presents a generic framework to assess life detection claims that, if followed, will increase confidence in exoplanet biosignature detection and interpretation.

The direct spectrum of a temperate terrestrial planet could also contain light reflected from its solid surface. In that case, surface biosignatures such as microbial pigments and/or the sharp increase in reflectivity of photosynthetic vegetation – the so-called red edge – could be detectable (e.g., Borges et al. 2024). However, detecting surface biosignatures requires very favorable circumstances: a small cloud–covering fraction and a large portion of the planet’s surface covered with life. Furthermore, potential false positives caused by non-living mineral pigments (e.g., iron oxide and iron hydroxide) must be carefully considered and ruled out.

### Surface Features, Magnetic Fields and Geodetic Observables

Perhaps surprisingly, it may be easier to detect biosignatures on an Earth-like exoplanet than to confirm the presence of *liquid* surface water. While the large amount of atmospheric water vapor absorption seen in the direct spectrum suggests the presence of liquid surface water, it is not conclusive evidence of oceans. Fortunately, obtaining many such spectra over time may allow one to recover the planet’s rotation period and reveal albedo inhomogeneities indicative of surface features such as clouds, continents, and oceans (e.g., Cowan et al. 2009; Berdyugina et al. 2019). Data from the Earth-observing satellite DSCOVR (Herman et al. 2018) have been used to show that even single–pixel observations of Earth (with high cadence) can provide a reasonably good estimate of the land/ocean mask and a planet’s rotation rate (e.g. Jiang et al. 2018; Fan et al. 2019).

Furthermore, the detection of ocean glint, which is caused by specular reflection off liquid water, may be possible. This has been demonstrated using EPOXI satellite observations of Earth (Livengood et al. 2011; Robinson et al. 2011). These observations also suggest that snowball Earth-type scenarios could be observable on terrestrial exoplanets as a wavelength-dependent change in the planet’s reflection spectrum compared to modern Earth (Cowan et al. 2011). The presence of distinct continents and oceans may provide indirect, tentative evidence of tectonics on a terrestrial exoplanet but is likely only feasible under favorable circumstances, such as a host system that is not too distant from the Sun and a small cloud coverage fraction on the planet.

Many attempts have been made to infer the presence of magnetic fields on gas giant exoplanets by searching for auroral emission. Searching for exoplanetary aurorae can take advantage of the fact that certain auroral features of giant planets, such as non-thermal radio emission (e.g., Grießmeier et al. 2007) and infrared $\mathrm{H}_{3}^{+}$ emission (Richey-Yowell et al. 2025), should not appear strongly in the spectra of the host stars, thus reducing the likelihood of false positives. Unfortunately, these attempts have not yet been clearly successful. A few tentative detections of radio emission from giant planets have been reported (e.g., Lecavelier des Etangs et al. 2013), although one of the most promising detections, $\tau $ Boötis b (Turner et al. 2021), was not confirmed in follow-up observations (Cordun et al. 2025).

Regarding terrestrial exoplanets, some calculations suggest that radio emission from such planets orbiting M dwarf stars is probably undetectable with current facilities under normal levels of stellar activity (Vidotto et al. 2019). However, others are more optimistic (e.g., Peña-Moñino et al. 2024). This theoretical debate is unlikely to be resolved without clear observational evidence. Regarding temperate terrestrial planets around Sun-like stars, it appears that much predictive theoretical work on potentially promising magnetic field signatures remains to be done.

Direct constraints on interior structure of exoplanets beyond mass and radius measurements which result in bulk densities, are scarce (e.g., Baumeister et al. 2025, this topical collection). In the Solar System, moment of inertia (MoI) measurements are a powerful tool for determining the interior density distributions of planets and moons, allowing estimates of core sizes. The MoI factor can be calculated from measurements of the gravitational moment, $J_{2}$, from the orbital motion of spacecraft or natural satellites (e.g., Iess et al. 2018) or from observations of the precession of the planet’s rotation axis (e.g., Folkner et al. 1997). However, even for Venus the MoI factor is poorly constrained (Margot et al. 2021). Unfortunately, the first type of measurement is not possible for exoplanets, and the second type would require high-quality direct spectra obtained over extremely long timescales. Closely related to the MoI factor are the fluid Love numbers $k_{n}$ and $h_{n}$ (Love 1911), which describe, respectively, the gravitational response and shape deformation of a planet of degree $n$ under the influence of a perturbing centrifugal or tidal potential. Of particular interest is the second–degree fluid Love number $k_{2}$. Assuming that the planet is in hydrostatic equilibrium, $k_{2}$ depends solely on the density distribution of the interior and $h_{2} = 1 + k_{2}$. Thus, measuring either $k_{2}$ or $h_{2}$ can allow us to meaningfully characterize the interior structure in principle, as discussed in e.g., Padovan et al. (2018), Baumeister et al. (2020), and Baumeister and Tosi (2023).

The shape deformation of a planet, which allows for the inference of $h_{2}$, can potentially be measured in transit light curves as a deviation from the light curve produced by a perfectly spherical planet (Hellard et al. 2019; Akinsanmi et al. 2019). If the planet is on an elliptical orbit and is close enough to its host star, the orbit will precess at a rate determined in part by the planet’s gravitational response, thus allowing an estimate of $k_{2}$ (Csizmadia, Hellard and Smith 2019). At present, $k_{2}$ has been measured for a few hot Jupiter exoplanets (Csizmadia, Hellard and Smith 2019; Barros et al. 2022; Bernabò et al. 2024, 2025). Such measurements will be much more difficult for rocky planets. Kálmán et al. (2025) estimate that oblateness values $f$ below that of Jupiter ($f\approx 0.06$) are not detectable with current transit photometry methods (see also Berardo and De Wit 2022). Therefore, measuring the oblateness of rocky planets, such as Earth with an oblateness of $f\approx 0.003$), is likely not possible in the foreseeable future. However, the ever-increasing time baseline of transit and radial velocity measurements may make measurements of Love numbers for sub-Jupiter planets possible in the coming decades.

### Technosignatures

Finally, we consider the detectability of technosignatures, the indirect signs of industry and/or technology. Many phenomena have been suggested as possible technosignatures, including narrowband radio signals (e.g., Tarter et al. 1983); waste heat (e.g., Wright et al. 2014); artificial illumination (e.g., Loeb and Turner 2012); and artificial atmospheric constituents, i.e., pollutants such as NO_2_ (Kopparapu et al. 2021). While these phenomena are familiar to us, we must acknowledge that they are extremely recent when compared to the approximately billions of years that life has existed on Earth. Detecting a phenomenon of short duration requires a large sample size. However, without knowing the duration, it is impossible to determine how large the sample size must be. Furthermore, the proposed technosignatures generally require massive magnifications of the source technology compared to Earth-analog levels to be detectable with current and planned facilities.

The limited, “1-D” data that we can collect for exoplanets in the foreseeable future also means that, as with biosignatures, there will be unknowns that complicate the interpretation of possible technosignatures. For instance, if artificial illumination is detectable at all, it could be mistaken for the planet’s unknown albedo in a secondary eclipse/phase curve observation, or it could be mistaken for a planet with an unusual scattering phase function during a direct observation (a false negative). One must also be wary of astrophysical objects that can mimic technology. A debris disk of interplanetary dust absorbing starlight and re-emitting it at infrared wavelengths, for instance, could be interpreted as a Kardashian Type II civilization giving off waste heat (a false positive; Wright et al. 2014). In sum, the prospects for detecting extraterrestrial technology, should it exist in our galactic neighborhood, do not appear promising. However, this possibility should be kept in mind as the characterization of alien worlds grows from a trickle to a flood in the coming decades.

## Exoplanet Science Lessons for the Geosciences

No subfield of planetary science rivals the wealth of data available for planet Earth. A wide range of geological and geophysical data exists for the planet, including its interior, atmosphere, oceans, biosphere, and space environment. This makes Earth the natural reference point for comparative planetology and space exploration. Space missions exploring the Solar System have revealed the diversity of its bodies. These include tidally heated moons with enormous heat flow and volcanic activity by Earth standards, ice-covered surfaces with subsurface oceans, diverse atmospheric compositions and surface rock lithologies, varying interior structures, tectonic and volcanic activity, and magnetic properties. Space exploration is the foundation of comparative planetology, a field through which geoscientists can learn to contextualize their home planet. Exploring the galaxy for exoplanets broadens our perspective and reveals even more diversity, particularly because the large number of planets will allow statistical studies. However, we have come to acknowledge that the Earth is special and that even the configuration of planets in the Solar System does not serve as a universal model.

The Earth and the planets of the Solar System are near the boundaries or outside the field of the currently known exoplanet populations, as shown in Fig. 1. While the radii overlap, it is mostly the orbital distance (and the type of central star) that makes the difference. This is certainly an observational bias that will improve with future observations such as those from the PLATO mission (e.g., Rauer et al. 2014).

Of the more than 6000 exoplanets discovered and confirmed to date (with about 8000 more awaiting confirmation), the NASA exoplanet archive exoplanetarchive.ipac.caltech.edu/docs/counts_detail.html (retrieved Dec 22, 2025) currently lists ∼1450 planets with radii ≤ 2 Earth radii, masses between 0.5 and 10 Earth masses, and estimated densities between 3000 and 6000 kg/m^3^ that may be rocky. Sixty-seven rocky planets are considered potentially habitable (Bohl et al. 2025), including the remarkable TRAPPIST-1 planetary system (see Ducrot et al. 2026 in this topical collection).

Some Earth-sized planets and most known super-Earths orbit their host planets “close in” and are tidally locked in 1:1 resonance. Due to strong insolation and tidal heating, these planets may possess (hemispheric) magma oceans (see Lustig-Yaeger et al. 2026 in this topical collection). These planets provide opportunities to study processes that are thought to have occurred on early Earth (e.g. Harrison 2020). Some of these magma–ocean planets are bright in the infrared and can be observed directly (e.g., 55 Cancri e; Hammond and Pierrehumbert 2017). Assuming the necessary data is available from future observations, other exoplanets can be used as analogues at other stages of Earth’s history. Similar to the Solar System and supported by planet formation theory, the age of the central star, as determined by stellar seismology, is considered equal to the age of the planets. This allows us to explore different evolutionary paths and improve theoretical models of planetary interiors, geodynamic regimes, atmospheres, oceans, life, and habitability.

### Diversity of Composition, Mineralogy, and Interior Processes

Transit and radial velocity exoplanet detection methods (e.g., Deeg and Alonso 2024; Trifonov 2026), and Transit Timing Variation observations (e.g., Agol and Fabrycky 2025) can be used to infer a planet’s radius and mass, respectively, or both (see Fig. 3). From these values, one can estimate the planet’s density and model its internal structure using, in addition, mineral physics data as discussed in Baumeister et al. 2025 in this topical collection. These models are aided by stellar spectroscopic data that provide constraints on the composition of the protoplanetary disk and can be used to infer a planet’s composition, within the limit of available thermodynamic databases (e.g., Hinkel et al. 2014; Putirka et al. 2021; Unterborn et al. 2018; Wang et al. 2022; Spaargaren et al. 2023). Furthermore, polluted white dwarfs can provide valuable information on the chemical composition of exoplanetary systems (Xu and Bonsor 2021). Notably, the current data significantly expand the range of compositions and mineralogies compared to the Solar System, in both the quartz-poor and the quartz–rich directions (e.g., Putirka et al. 2021).

Rocky exoplanets are often assumed to have characteristics similar to those of Earth and the other planets in the Solar System. Specifically, they are thought to have a structure consisting of a metallic iron-dominated core, a silicate mantle, and a basalt–like crust. However, some exoplanets have densities close to that of iron suggesting high proportions of heavy elements and large cores (see Fig. 3). These are termed super-Mercuries.^13^ Others have densities between MgSiO_3_ and water ice, suggesting a high content of volatiles, and are termed super-Ganymedes.^14^ Sub-Neptunes have low densities and are thought to have Earth-like rocky cores and thick atmospheric envelopes. Finally, super-Earths, which were already referred to above, are planets with Earth-like densities but radii a few times larger. This wide range of planetary sizes and masses results in vastly different properties regarding pressure, temperature, thermal and geodynamic evolution, and, possibly, ultimately habitability (Balmer and Noack 2021; Dorn et al. 2018). For instance, the pressure in the deep mantle and core of a super-Earth can reach values of terapascals (1 TPa = 1000 GPa) and the temperature can exceed 5000 K (e.g., Stamenković et al. 2012). At such high pressures, minerals can crystallize into highly compact structures with significantly different physical properties than the lower-pressure phases that dominate the Earth’s mantle (e.g., Dutta et al. 2022; Zurkowski et al. 2022; Stamenković et al. 2011; Karato 2011). These properties may affect mantle convection, thermal evolution, and core dynamo action.

**Fig. 10:**
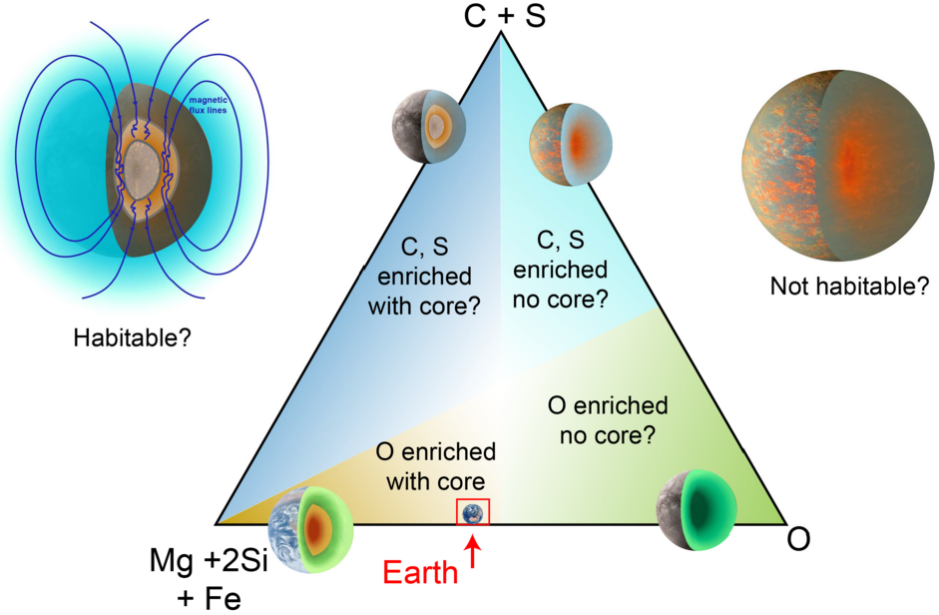
Schematic view of the compositional space spanned by the three major planet forming elements: 1) magnesium, silicate and iron, 2) oxygen, and 3) carbon and sulfur.(Modified from Unterborn et al. 2014.) The composition of Earth is also shown. Differences in composition can lead to significantly different interior structures, as oxygen-bearing minerals are replaced by carbon- and sulfur–bearing minerals. Differences in the physio-chemical properties of minerals can lead to significant difference in a planet’s large–scale properties. Planets near the Mg+2Si+Fe-rich apex will likely differentiate and form a core, resembling Earth. O– and C+S– rich planets may be undifferentiated, but they have received little study

The interior chemistry of a planet also plays a pivotal role in its evolution. In recent years, observations of carbon-enriched stars have prompted numerous studies on carbon-enriched planets (e.g., Nisr et al. 2017; Daviau and Lee 2018), some of which have been expanded to include sulfur as well (e.g., Hakim et al. 2019). Interest in studying mineralogies with abundant carbon and sulfur also stems from spacecraft observations of Mercury and Venus. In oxygen-poor environments, the major elements that form planets can bond with carbon and sulfur rather than with oxygen, resulting in reduced carbon- and sulfur-bearing phases. These phases have also been observed in certain geological contexts on Earth, as well as in extraterrestrial materials such as presolar grains and meteorites. Chemical diversity can lead to substantial diversity in planetary properties and interior structure as illustrated in Fig. 10. See, for example, Unterborn et al. (2014) and van Hoolst et al. (2019).

Carbon and carbon–bearing phases such as silicon-carbide (SiC) are less dense than typical mantle silicates. Therefore, in the presence of a mixed mantle – as suggested by Schmidt et al. (2014) for reducing conditions – it would be possible for these minerals to upwell and form a carbon–rich upper layer. A similar mechanism has been proposed as the origin of the floating graphite crust on Mercury (Peplowski et al. 2016; Keppler and Golabek 2019). Miozzi et al. (2018) conducted numerical simulations of an archetypal SiC mantle and demonstrated that SiC’s distinct properties relative to silicates render the mantle less susceptible to convection. However, the simulations do not depict layered convection, as suggested by Nisr et al. (2017). Unfortunately, the potential dynamic regime of reduced planets has received little study and many of the characteristics of these potential mantles remain unknown.

Sulfur-bearing materials have received more attention as potential components of Mercury’s mantle. For instance, Pommier et al. (2023) report the formation of Mg-S and Ca-S bonds in glasses under highly reducing conditions, while Mouser et al. (2021) investigate end-member scenarios for mantle evolution based on experimentally collected viscosity data for a Mercury-like magma ocean. Changes in the ratio and chemistry of a planet’s major elemental components would affect its composition and, in the extreme scenarios, whether it has a core. The Fe-S-C system exhibits an immiscibility gap at lower pressures, leading to the formation of two distinct metallic phases (e.g., Hakim et al. 2019). However, the evolution of the ternary system at pressures and temperatures relevant to Earth-sized planets, has not been investigated. Understanding core composition and structure is fundamental to assessing the possibility of a geodyamo (see Lourenço et al. 2026 in this topical collection).

Directly imaging the surface of exoplanets with sufficient resolution to distinguish geodynamic regimes will be difficult in the foreseeable future. However, we may be able to infer their tectonics using indirect observations, such as their atmospheric compositions and potential magnetic fields. Gases released into the atmosphere by volcanism provide insight into a planet’s geodynamics. On Earth, for example, the composition of the atmosphere is a consequence of the (bio)geochemical cycles maintained by the current regime of plate tectonics (Schaefer and Parmentier 2021). Advanced Earth System modeling tools will improve our understanding of the range of possible geodynamic regimes and those that Earth has passed through. Improved observations of Earth-like planets at different stages of evolution may also offer direct insights into Earth’s past and future. Coupling observations with theoretical models may improve our understanding of planetary geodynamics and the conditions necessary for plate tectonics. Larger planets may be able to retain more heat resulting in more vigorous mantle convection. However, higher thermal energy could potentially prevent the formation of a strong lithosphere. This suggests that a delicate balance is needed to maintain plate tectonics (e.g., O’Neill et al. 2007; Korenaga 2010; Foley et al. 2012; Noack and Breuer 2014). Other factors, such as intense solar radiation and the presence or absence of water (and even life), are thought to play a fundamental role in permitting or preventing plate tectonics. Examining the library of exoplanets will allow us to improve our understanding of how wide (or narrow) this balance is. This will be key to understanding why Earth developed plate tectonics and the main factors controlling it.

### Atmospheres and Oceans

The study of exoplanets reveals a wide range of atmospheric compositions and potential climates (Schaefer and Parmentier 2021). These compositions depend on a planet’s mass, composition, geodynamic regime, and external processes, such as photodissociation and atmospheric erosion. The range of orbital parameters and planetary sizes also strongly affects their atmospheric circulation and climatic patterns. Studying Earth-like exoplanets in various stages of evolution improves our understanding of processes such as the formation of primitive atmospheres, atmospheric escape, and the formation of clouds and hazes (Zahnle and Catling 2017; Fauchez et al. 2019). James Webb Space Telescope observations will also contribute to our understanding of photochemical processes within exoplanetary atmospheres, which are key to identifying biosignatures.

Data from transit spectroscopy and secondary eclipses will reveal a variety of atmospheric compositions and improve our climate models (Komacek et al. 2021). Investigating a wider range of conditions will help us assess the robustness and predictability of our current global circulation models of past and future Earth (e.g., Way et al. 2021). This information is crucial for understanding how the Earth’s atmosphere and climate have changed over time and what future pathways may be possible. Studying exoplanets that have undergone runaway greenhouse effects or lost their atmospheres will help us identify critical thresholds that trigger these extreme (and potentially irreversible) climate states (e.g., Chaverot et al. 2023; Airapetian et al. 2020; Way and Del Genio 2020). Additionally, since exoplanet atmospheres will be our only “direct” window to their (bio)geodynamics for the foreseeable future, understanding how they operate is crucial to improving our knowledge of planetary geodynamics with sufficient precision to apply it to the past and future of Earth.

Studies of exoplanets have suggested the presence of exo-oceans in the form of subsurface oceans in ice worlds and deep, global surface oceans in aquaplanets (e.g., Cowan et al. 2009; Cowan and Abbot 2014; Piaulet et al. 2023). Observing exo-oceans will provide new insights into our own ocean, particularly how it formed and evolved (see Sect. 3). There are uncertainties about the sources of water that led to the formation of the Earth’s oceans (e.g., Karato 2015). For example, we do not know how much water primitive Earth had and how much was brought from the outside by comets or chondrites (e.g. Marty 2012). Future direct observations of magma ocean worlds could provide valuable information about degassing processes and interactions between a magma ocean and the atmosphere (e.g. Way et al. 2023; Salvador et al. 2023). Furthermore, observing exo-oceans with different depths and compositions (e.g., ammonia and hydrocarbons) may help us understand the likelihood of forming an Earth-like ocean and maintaining it. Understanding how ocean diversity impacts geochemical cycling, climate, and plate tectonics is also important. This knowledge will improve our understanding of how these processes have operated throughout Earth’s history. For now, part of this work must be based on modeling, but future observations will be crucial to validating these models.

### Life

The search for life beyond Earth remains the primary motivation for extraterrestrial research. This applies to searches within and beyond the Solar System. Life on Earth can exist in a wide range of conditions, from deep within the crust to high in the atmosphere^15^ (e.g., Schulze-Makuch and Irwin 2018) and it comes in a multitude of species. However, extraterrestrial life is expected to offer its own surprises. As we concluded in previous sections of this article, finding a second Earth with *life-as-we-know-it* appears challenging if not unlikely. The first inhabited planet discovered will likely not be another Earth! While this conclusion complicates the search for biosignatures, a positive result would be all the more exciting and rewarding for our understanding of life on Earth.

## Concluding Thoughts on the Perspectives of Exo-Geoscience

Exo-geoscience is an emerging field that brings together geoscientists, astrophysicists, planetary scientists, and astrobiologists (e.g., Unterborn et al. 2020; Shorttle et al. 2021). Geoscientists play a pivotal role in helping to interpret exoplanet observations by offering insights from Earth. In the process, they gain valuable insights about Earth by comparing it to discoveries on other worlds. Earth and the other rocky planets and moons in the Solar System continue to serve as *the* reference case for potential discoveries in other planetary systems throughout the galaxy and beyond. However, it is important to understand that the Solar System does not represent a unique pathway for planetary evolution. Due to the dearth of data that will likely persist into the foreseeable future, we must be very cautious when drawing conclusions about extrasolar terrestrial planets.

As we have noted, Earth is a living planet that has co-evolved with life, possibly after passing through early bottlenecks for life by chance (Lineweaver and Chopra 2025). These bottlenecks may have been climatic or related to early meteoritic bombardment or due to volcanic activity on the planet. Earth’s location at the right distance from the Sun and in the right corner of the galaxy made it possible for life to emerge. One could argue that Venus and Mars may have developed life early on, but that their orbital distances may not have been quite as favorable. Thus, answering the open question of whether there is extant or extinct life on Mars, and less likely perhaps on Venus, is important. However, one could argue that this question can only be definitively answered by detecting life on these planets. Continued failure to detect life may just keep the question open.

There is good reason to argue that *life-as-we-know-it* on Earth depends on the properties of this planet. These properties include plate tectonics, which maintains clement climate conditions, renews basic nutrients, sustains bio-relevant geochemical cycles, maintains a diverse environment with land and oceans, and a magnetic field. It is also being increasingly recognized that life plays a role in the global functioning of Earth as an inhabited, habitable planet. Considering the evolution of life on Earth, it is becoming increasingly clear that it is not simply the living planet moving through the evolutionary landscape on an optimal path. Rather the evolution of the biosphere to consciousness and the ability to explore its cosmic neighborhood and answer the questions posed in this article may be due to contingency in both the geological and cultural evolution. However, it is essential to remain open to other realizations of inhabited planets.

For geoscientists, interest in exoplanetary science is largely motivated by the large number of planets that may eventually allow for statistical analysis. As we have described above, exploring the Solar System has provided some insight into planetary diversity. Exploring exoplanets will provide a much wider range of planetary states that can help to improve our understanding of the Earth (Meadows et al. 2018; Lammer et al. 2009). Detecting planets in different stages of evolution will offer insights into how Earth may have been in the past and how it evolved, by studying processes that no longer occur on our planet. However, since direct observations are limited, we will need to enhance our theoretical models and laboratory studies of planetary material properties. The databases currently available and used to investigate processes on Earth need to be expanded to account for those phases that are not common or existing on Earth, but can still belong to similarly defined chemical systems and are observed in extraterrestrial materials. The pressure and temperature range in which the physiochemical properties of minerals are determined need to be expanded. At the same time, a more comprehensive database of transport properties should be built. The latter are extremely challenging to determine experimentally and require the development of new techniques. Another frontier for experimental work is the study of hydrogen and its interaction with melts, minerals, and metals, representative of the magma oceans and planetary mantles and cores respectively. Mineral properties lie at the base of every process occurring on planets and as such, it is fundamental to have the most complete possible characterization in order to model planetary processes. And a new generation of models must integrate the various components of the planetary system (Foley and Driscoll 2016). The current momentum in exoplanet research will certainly result in a much improved understanding of the Earth. Lessons learned from studying exoplanets will help us distinguish the universal properties of rocky planets from contingent ones and the results of chance.

## Footnotes

1 The circumstellar habitable zone is the annular region around a star where the physical conditions allow the existence of liquid water on the surface of a rocky planet (e.g., Lingam and Balbi 2024, Sect. 8.1). It was first introduced by Hart (1979) and quantified by Kasting et al. (1993).

2 The Copernican Principle states that Earth does not occupy a special or privileged position in the universe.

3 The Principle of Mediocrity states that Earth is not special or unique within the cosmos.

4 The galactic habitable zone GHZ (e.g., Gowanlock et al. 2011; Lineweaver 2025) is defined as the region of space and time within a galaxy that is compatible with life-as-we-know-it. These regions contain main-sequence stars that are relatively far from life-extinguishing supernovae. Main-sequence stars in the GHZ have sufficient metallicity to allow the formation of rocky planets, yet not enough to produce giant planets that can migrate through the circumstellar habitable zone. The main-sequence lifetimes of these stars should allow for the formation and evolution of life.

5 We distinguish between intelligent life and life with technological intelligence, since animals can possess intelligence.

6 Molecular oxygen is highly reactive, so the reduced organic compounds necessary for the origin of life would be degraded by oxidation (e.g., Deamer et al. 2022; Rimmer et al. 2021).

7 Net primary productivity (NPP) is the rate at which energy (carbon) is stored as biomass by plants and other primary producers in an ecosystem.

8 Note that it may not have even been the largest recorded impactor in Earth’s history (e.g. Sleep and Lowe 2014), omitting the Moon forming impact of course (e.g. Benz et al. 1986).

9 Contingency refers to an outcome that is decisively influenced by unpredictable circumstances and chance. Convergence, or determinism, describes the tendency for similar forms or solutions to arise independently in different places due to common constraints or similar environmental pressures.

10 https://www.esa.int/Science_Exploration/Space_Science/Ariel_factsheet.

11 https://elt.eso.org.

12 https://science.nasa.gov/astrophysics/programs/habitable-worlds-observatory/.

13 For being presumably close in composition to, but larger than, Mercury.

14 For being presumably close in composition to, but larger than, the Jovian moon Ganymede.

15 Self-replication of life in the atmosphere has yet to be observed (e.g. Tastassa et al. 2024).

## References

[CR1] Acuña M, Connerney J, Ness, et al. (1999) Global distribution of crustal magnetization discovered by the Mars Global Surveyor MAG/ER experiment. Science 284(5415):790–793. 10.1126/science.284.5415.790 10221908 10.1126/science.284.5415.790

[CR2] Adams FC (2010) The birth environment of the Solar System. Annu Rev Astron Astrophys 48(1):47–85. 10.1146/annurev-astro-081309-130830

[CR3] Agol E, Fabrycky D (2025) Transit timing and duration variations for the discovery and characterization of exoplanets in the TESS era. arXiv:1706.09849

[CR4] Airapetian V, Barnes R, Cohen O, et al. (2020) Impact of space weather on climate and habitability of terrestrial-type exoplanets. Int J Astrobiol 19(2):136–194. 10.1017/S1473550419000132

[CR5] Akinsanmi B, Barros SCC, Santos NC, et al. (2019) Detectability of shape deformation in short-period exoplanets. Astron Astrophys 621:A117. 10.1051/0004-6361/201834215

[CR6] Alcott LJ, Mills BJ, Poulton SW (2019) Stepwise Earth oxygenation is an inherent property of global biogeochemical cycling. Science 366(6471):1333–1337. 10.1126/science.aax64 31826958 10.1126/science.aax6459

[CR7] Alcott LJ, Mills BJ, Bekker A, et al. (2022) Earth’s great oxidation event facilitated by the rise of sedimentary phosphorus recycling. Nat Geosci 15(3):210–215. 10.1038/s41561-022-00906-5

[CR8] Alvarez LW, Alvarez W, Asaro F, et al. (1980) Extraterrestrial cause for the Cretaceous-Tertiary extinction. Science 208(4448):1095–1108. 10.1126/science.208.4448.1095 17783054 10.1126/science.208.4448.1095

[CR9] Anderson BJ, Acuña MH, Korth H, et al. (2010) The magnetic field of Mercury. Space Sci Rev 152(1):307–339. 10.1007/s11214-009-9544-3

[CR10] Apai D, Barnes R, Murphy MM, et al. (2025) A terminology and quantitative framework for assessing the habitability of Solar System and extraterrestrial worlds. Planet Sci J 6:165. 10.3847/PSJ/addd8

[CR11] Awramik SM, McNamara KJ (2007) The evolution and diversification of life. In: Sullivan WT, Barros JA (eds) Planets and life. Cambridge University Press, Cambridge, pp 313–334

[CR12] Baines KH, Atreya SK, Bullock MA, et al.(2013) The atmospheres of the terrestrial planets: Clues to the origins and early evolution of Venus, Earth, and Mars. In: Mackwell SJ, et al.(ed) Comparative climatology of terrestrial planets, University of Arizona Press, Tucson, pp 137–160

[CR13] Balbi A, Lingam M (2023) Beyond mediocrity: how common is life? Mon Not R Astron Soc 522(2):3117–3123. 10.1093/mnras/stad1155

[CR14] Balmer MD, Noack L (2021) The diversity of exoplanets: from interior dynamics to surface expressions. Elements 17(4):245–250. 10.2138/gselements.17.4.245

[CR15] Bar-On YM, Phillips R, Milo R (2018) The biomass distribution on Earth. Proc Natl Acad Sci USA 115(25):6506–6511. 10.1073/pnas.1711842115 29784790 10.1073/pnas.1711842115 PMC6016768

[CR16] Barros SCC, Akinsanmi B, Boué G, et al. (2022) Detection of the tidal deformation of WASP-103b at 3 with CHEOPS. Astron Astrophys 657:A52. 10.1051/0004-6361/202142196

[CR17] Basalla G (2006) Civilized life in the universe: scientists on intelligent extraterrestrials. Oxford University Press, Oxford. 10.1093/acprof:oso/9780195171815.002.0003

[CR18] Baumeister P, Tosi N (2023) ExoMDN: rapid characterization of exoplanet interior structures with mixture density networks. Astron Astrophys 676:A106. 10.1051/0004-6361/202346216

[CR19] Baumeister P, Padovan S, Tosi N, et al. (2020) Machine-learning inference of the interior structure of low-mass exoplanets. Astrophys J 889(1):42. 10.3847/1538-4357/ab5d32

[CR20] Baumeister P, Miozzi F, Guimond CM, et al. (2025) Fundamentals of interior modelling and challenges in the interpretation of observed rocky exoplanets. Space Sci Rev 221(8):123. 10.1007/s11214-025-01248-5 41356077 10.1007/s11214-025-01248-5 PMC12675577

[CR21] Benz W, Slattery W, Cameron A (1986) The origin of the Moon and the single-impact hypothesis I. Icarus 66(3):515–535. 10.1016/0019-1035(86)90088-6 10.1016/0019-1035(89)90129-2 11542164

[CR22] Berardo D, De Wit J (2022) On the effects of planetary oblateness on exoplanet studies. Astrophys J 935(2):178. 10.3847/1538-4357/ac82b2

[CR23] Berdyugina SV, Kuhn JR, Belikov R, et al. (2019) Exoplanet terra incognita. In: Hargitai H (ed) Planetary cartography and GIS. Springer, Cham, pp 337–351. 10.1007/978-3-319-62849-3_20

[CR24] Bernabò LM, Sz C, Smith AMS, et al. (2024) Evidence of apsidal motion and a possible co-moving companion star detected in the WASP-19 system. Astron Astrophys 684:A78. 10.1051/0004-6361/202346852

[CR25] Bernabò LM, Sz C, Smith AMS, et al. (2025) Characterising WASP-43b’s interior structure: unveiling tidal decay and apsidal motion. Astron Astrophys 694:A233. 10.1051/0004-6361/202451994

[CR26] Berner RA (2006) Geological nitrogen cycle and atmospheric N_2_ over Phanerozoic time. Geology 34(5):413–415. 10.1130/G22470.1

[CR27] Black BA, Karlstrom L, Mills BJ, et al. (2024) Cryptic degassing and protracted greenhouse climates after flood basalt events. Nat Geosci 17(11):1162–1168. 10.1038/s41561-024-01574-3

[CR28] Blackman EG, Tarduno JA (2018) Mass, energy, and momentum capture from stellar winds by magnetized and unmagnetized planets: implications for atmospheric erosion and habitability. Mon Not R Astron Soc 481(4):5146–5155. 10.1093/mnras/sty2640

[CR29] Blount ZD, Lenski RE, Losos JB (2018) Contingency and determinism in evolution: replaying life’s tape. Science 362(6415):eaam5979. 10.1126/science.aam5979 30409860 10.1126/science.aam5979

[CR30] Bohl A, Lawrence L, Lowry G, et al (2025) Probing the limits of habitability: a catalog of rocky exoplanets in the habitable zone. arXiv:2501.14054

[CR31] Bond DP, Grasby SE (2017) On the causes of mass extinctions. Palaeogeogr Palaeoclimatol Palaeoecol 478:3–29. 10.1016/j.palaeo.2016.11.005.

[CR32] Bond D, Wignall P, Keller G, et al. (2014) Volcanism, impacts, and mass extinctions: causes and effects. Spec Pap, Geol Soc Am 505:29–55. 10.1130/9780813725055

[CR33] Bondi H (1952) Cosmology. Cambridge University Press, Cambridge

[CR34] Borges SR, Jones GG, Robinson TD (2024) Detectability of surface biosignatures for directly imaged rocky exoplanets. Astrobiology 24(3):283–299. 10.1089/ast.2023.0099 38377582 10.1089/ast.2023.0099

[CR35] Bower DJ, Hakim K, Sossi PA, et al. (2022) Retention of water in terrestrial magma oceans and carbon-rich early atmospheres. Planet Sci J 3(4):93. 10.3847/PSJ/ac5fb1

[CR36] Breuer D, Spohn T (2023) Terrestrial planets: interior structure, dynamics, and evolution. Oxford research encyclopedia of planetary science. Oxford University Press, Oxford. 10.1093/acrefore/9780190647926.013.28

[CR37] Bryson JF, Nichols CI, Herrero-Albillos J, et al. (2015) Long-lived magnetism from solidification-driven convection on the pallasite parent body. Nature 517(7535):472–475. 10.1038/nature14114 25612050 10.1038/nature14114

[CR38] Byrne P, Guimond CM, Cawood P, et al (2026) What the Solar System can teach us about rocky exoplanets. Space Sci Rev. Submitted 10.1007/s11214-026-01325-3 PMC13562164 42729352

[CR39] Catling DC, Zahnle KJ (2020) The Archean atmosphere. Sci Adv 6(9):eaax1420. 10.1126/sciadv.aax1420 32133393 10.1126/sciadv.aax1420 PMC7043912

[CR40] Cawood PA, Hawkesworth CJ (2019) Continental crustal volume, thickness and area, and their geodynamic implications. Gondwana Res 66:116–125. 10.1016/j.gr.2018.11.001

[CR41] Cawood PA, Chowdhury P, Mulder JA, et al. (2022) Secular evolution of continents and the Earth system. Rev Geophys 60(4):e2022RG000789. 10.1029/2022RG000789

[CR42] Chambers J (2020) The effect of seafloor weathering on planetary habitability. Astrophys J 896(2):96. 10.3847/1538-4357/ab94a4

[CR43] Charnay B, Le Hir G, Fluteau F, et al. (2017) A warm or a cold early Earth? New insights from a 3-D climate-carbon model. Earth Planet Sci Lett 474:97–109. 10.1016/j.epsl.2017.06.029

[CR44] Chaverot G, Bolmont E, Turbet M (2023) First exploration of the runaway greenhouse transition with a 3D general circulation model. Astron Astrophys 680:A103. 10.1051/0004-6361/202346936

[CR45] Chopra A, Lineweaver CH (2016) The case for a Gaian bottleneck: the biology of habitability. Astrobiology 16(1):7–22. 10.1089/ast.2015.1387 26789354 10.1089/ast.2015.1387

[CR46] Chowdhury P, Cawood PA, Mulder JA (2025) Subaerial emergence of continents on Archean Earth. Annu Rev Earth Planet Sci 53:443–478. 10.1146/annurev-Earth-040722-093345

[CR47] Christensen UR (2010) Dynamo scaling laws and application to the planets. Space Sci Rev 152:565–590. 10.1007/s11214-009-9553-2

[CR48] Christensen UR, Holzwarth V, Reiners A (2009) Dynamo scaling laws and application to the planets. Nature. 457:167–169. 10.1038/nature07626 19129842 10.1038/nature07626

[CR49] Claire MW, Sheets J, Cohen M, et al. (2012) The evolution of solar flux from 0.1 nm to 160 m: quantitative estimates for planetary studies. Astrophys J 757(1):95. 10.1088/0004-637X/757/1/95

[CR50] Clery D (2023) Future NASA scope would find life on alien worlds. Science 379(6628):123–124. 10.1126/science.adg6273 36634175 10.1126/science.adg6273

[CR51] Cockell CS, Bush T, Bryce C, et al. (2016) Habitability: a review. Astrobiology 16(1):89–117. 10.1089/ast.2015.1295 26741054 10.1089/ast.2015.1295

[CR52] Cordun CM, Vedantham HK, Brentjens MA, et al. (2025) Deep radio interferometric search for decametre radio emission from the exoplanet Tau Boötis b. Astron Astrophys 693:A162. 10.1051/0004-6361/202452868. arXiv:2501.06301 [astro-ph.EP]

[CR53] Cowan NB, Abbot DS (2014) Water cycling between ocean and mantle: super-earths need not be waterworlds. Astrophys J 781(1):27. 10.1088/0004-637X/781/1/27. arXiv:1401.0720

[CR54] Cowan NB, Agol E, Meadows VS, et al. (2009) Alien maps of an ocean-bearing world. Astrophys J 700(2):915. 10.1088/0004-637X/700/2/915

[CR55] Cowan NB, Robinson T, Livengood TA, et al. (2011) Rotational variability of Earth’s polar regions: implications for detecting snowball planets. Astrophys J 731(1):76. 10.1088/0004-637X/731/1/76

[CR56] Csizmadia S, Hellard H, Smith AMS (2019) An estimate of the k_2_ love number of WASP-18Ab from its radial velocity measurements. Astron Astrophys 623:A45. 10.1051/0004-6361/201834376

[CR57] Daviau K, Lee KK (2018) High-pressure, high-temperature behavior of silicon carbide: a review. Crystals 8(5):217. 10.3390/cryst8050217

[CR58] De Duve C (2011) Life as a cosmic imperative? Philos Trans R Soc A, Math Phys Eng Sci 369(1936):620–623. 10.1098/rsta.2010.0312 10.1098/rsta.2010.0312 21220285

[CR59] Deamer D, Cary F, Damer B (2022) Urability: a property of planetary bodies that can support an origin of life. Astrobiology 22(7):889–900. 10.1089/ast.2021.0173 35675644 10.1089/ast.2021.0173

[CR60] Deeg HJ, Alonso R (2024) Transit photometry as an exoplanet discovery method. arXiv:1803.07867

[CR61] Desjardins E (2011) Historicity and experimental evolution. Biol Philos 26:339–364. 10.1007/s10539-011-9256-4

[CR62] Diamond J (1997) Guns, Germs and Steel: the fates of human societies. W. W. Norton, New York

[CR63] Dorn C, Noack L, Rozel A (2018) Outgassing on stagnant-lid super-earths. Astron Astrophys 614:A18. 10.1051/0004-6361/201731513

[CR64] Ducrot E, Bolmont E, Lustig-Yaeger J, et al (2026) TRAPPIST-1: a natural laboratory for the study of temperate rocky exoplanets. Space Sci Rev 222

[CR65] Durham WH (1991) Coevolution: genes, culture and human diversity. Stanford University Press, Stanford

[CR66] Dutta R, Tracy SJ, Cohen RE, et al. (2022) Ultra-high pressure disordered eight-coordinated phase of Mg_2_GeO_4_: analogue for super-Earth mantles. Proc Natl Acad Sci USA 119(8):e2114424119. 10.1073/pnas.2114424119 35165195 10.1073/pnas.2114424119 PMC8872715

[CR67] Dziewonski A, Rabinowicz B, Schubert G (eds) (2015) Treatise on geophysics. Deep Earth seismology, 2nd edn. vol 1. Elsevier, New York

[CR68] Ernst RE, Bleeker W, Söderlund U, et al. (2013) Large igneous provinces and supercontinents: toward completing the plate tectonic revolution. Lithos 174:1–14. 10.1016/j.lithos.2013.02.017

[CR69] Falkowski PG, Fenchel T, Delong EF (2008) The microbial engines that drive Earth’s biogeochemical cycles. Science 320(5879):1034–1039. 10.1126/science.1153213 18497287 10.1126/science.1153213

[CR70] Fan S, Li C, Li JZ, et al. (2019) Earth as an exoplanet: a two-dimensional alien map. Astrophys J Lett 882(1):L1. 10.3847/2041-8213/ab3a49

[CR71] Fauchez TJ, Turbet M, Villanueva GL, et al. (2019) Impact of clouds and hazes on the simulated JWST transmission spectra of habitable zone planets in the TRAPPIST-1 system. Astrophys J 887(2):194. 10.3847/1538-4357/ab5862

[CR72] Feinberg G, Shapiro R (1980) Life beyond Earth: the intelligent Earthling’s guide to life in the universe. William, Morrow

[CR73] Feulner G, Bukenberger M, Petri S (2023) Tracing the snowball bifurcation of aquaplanets through time reveals a fundamental shift in critical-state dynamics. Earth Syst Dyn 14(3):533–547. 10.5194/esd-14-533-2023

[CR74] Foley BJ, Driscoll PE (2016) Whole planet coupling between climate, mantle, and core: implications for rocky planet evolution. Geochem Geophys Geosyst 17:1885–1914. 10.1002/2015GC006210

[CR75] Foley BJ, Smye AJ (2018) Carbon cycling and habitability of Earth-size stagnant lid planets. Astrobiology 18(7):873–896. 10.1089/ast.2017.1695. arXiv:1712.03614 30035642 10.1089/ast.2017.1695

[CR76] Foley BJ, Bercovici D, Landuyt W (2012) The conditions for plate tectonics on super-Earths: inferences from convection models with damage. Earth Planet Sci Lett 331:281–290. 10.1016/j.epsl.2012.03.028

[CR77] Folkner WM, Yoder CF, Yuan DN, et al. (1997) Interior structure and seasonal mass redistribution of Mars from radio tracking of Mars Pathfinder. Science 278(5344):1749–1752. 10.1126/science.278.5344.1749 9388168 10.1126/science.278.5344.1749

[CR78] Förster MW, Foley SF, Alard O, et al. (2019) Partitioning of nitrogen during melting and recycling in subduction zones and the evolution of atmospheric nitrogen. Chem Geol 525:334–342. 10.1016/j.chemgeo.2019.07.042

[CR79] Genova A, Goossens S, Mazarico E, et al. (2019) Geodetic evidence that Mercury has a solid inner core. Geophys Res Lett 46(7):3625–3633. 10.1029/2018GL081135 31359894 10.1029/2018GL081135 PMC6662718

[CR80] Gerya T (2022) Numerical modeling of subduction: state of the art and future directions. Geosphere 18(2):503–561. 10.1130/GES02416.1

[CR81] Glaser DM, Hartnett HE, Desch SJ, et al. (2020) Detectability of life using oxygen on pelagic planets and water worlds. Astrophys J 893:163. 10.3847/1538-4357/ab822d

[CR82] Glaser DM, Olson S, Berdyugina S, et al (2026) Euhabitability: a new term to aid in the search for life in addition to the habitable zone framework. Space Sci Rev 222 10.1007/s11214-026-01315-5 PMC13447492 42568607

[CR83] Goldblatt C, Claire MW, Lenton TM, et al. (2009) Nitrogen-enhanced greenhouse warming on early Earth. Nat Geosci 2(12):891–896. 10.1038/ngeo692

[CR84] Goldblatt C, McDonald VL, McCusker KE (2021) Earth’s long-term climate stabilized by clouds. Nat Geosci 14(3):143–150. 10.1038/s41561-021-00691-7

[CR85] Goodwin AM (1996) Principles of Precambrian geology. Academic Press, London. 10.1016/B978-0-12-289770-2.X5000-6

[CR86] Gough DO (1981) Solar interior structure and luminosity variations. Sol Phys 74(1):21–34. 10.1007/BF00151270

[CR87] Gould SJ (1989) Wonderful life: the burgess shale and the nature of history. WW Norton & Company

[CR88] Gowanlock MG, Patton DR, McConnell SM (2011) A model of habitability within the Milky Way galaxy. Astrobiology 11(9):855–873. 10.1089/ast.2010.0555 22059554 10.1089/ast.2010.0555

[CR89] Greene TP, Bell TJ, Ducrot E, et al. (2023) Thermal emission from the Earth-sized exoplanet TRAPPIST-1b using JWST. Nature 618(7963):39–42. 10.1038/s41586-023-05951-7. arXiv:2303.14849 [astro-ph.EP] 36972683 10.1038/s41586-023-05951-7

[CR90] Gregg TK (2015) Planetary tectonics and volcanism: the inner Solar System. In: Spohn T, Schubert G (eds) Treatise on geophysics, 2nd edn. vol 10. Elsevier, New York, pp 307–326

[CR91] Grenfell JL (2017) A review of exoplanetary biosignatures. Phys Rep 713:1–17. 10.1016/j.physrep.2017.08.003

[CR92] Grießmeier JM, Zarka P, Spreeuw H (2007) Predicting low-frequency radio fluxes of known extrasolar planets. Astron Astrophys 475(1):359–368. 10.1051/0004-6361:20077397. arXiv:0806.0327 [astro-ph]

[CR93] Grießmeier J, Tabata-Vakili F, Stadelmann A, et al. (2016) Galactic cosmic rays on extrasolar Earth-like planets II. Atmospheric implications. Astron Astrophys 587:A159. 10.1051/0004-6361/201425452

[CR94] Guimond CM, Spohn T, Berdyugina S, et al. (2026) Water versus land on temperate rocky planets. Space Sci Rev 222:8. 10.1007/s11214-025-01264-5 41523890 10.1007/s11214-025-01264-5 PMC12783251

[CR95] Gunell H, Maggiolo R, Nilsson H, et al. (2018) Why an intrinsic magnetic field does not protect a planet against atmospheric escape. Astron Astrophys 614:L3. 10.1051/0004-6361/201832934

[CR96] Hakim K, Spaargaren R, Grewal DS, et al. (2019) Mineralogy, structure, and habitability of carbon-enriched rocky exoplanets: a laboratory approach. Astrobiology 19(7):867–884. 10.1089/ast.2018.193 30994366 10.1089/ast.2018.1930

[CR97] Hamano K, Abe Y, Genda H (2013) Emergence of two types of terrestrial planet on solidification of magma ocean. Nature 497:607–610. 10.1038/nature12163 23719462 10.1038/nature12163

[CR98] Hammond M, Pierrehumbert RT (2017) Linking the climate and thermal phase curve of 55 Cancri e. Astrophys J 849(2):152. 10.3847/1538-4357/aa9328

[CR99] Haqq-Misra JD, Domagal-Goldman SD, Kasting P, et al. (2008) A revised, hazy methane greenhouse for the Archean Earth. Astrobiology 8(6):1127–1137. 10.1089/ast.2007.0197 19093801 10.1089/ast.2007.0197

[CR100] Harris LB, Bedard JH (2014) Crustal evolution and deformation in a non-plate tectonic archaean Earth: comparisons with Venus. In: Dilek Y, Furnes H (eds) Evolution of archean crust and early life. Modern approaches in solid Earth sciences, vol 7. Springer, Dordrech, pp 215–288 10.1007/978-94-007-7615-9_9

[CR101] Harrison TM (2020) Hadean Earth. Springer, Cham. 10.1007/978-3-030-46687-9

[CR102] Hart MH (1979) Habitable zones about main sequence stars. Icarus 37(1):351–357. 10.1016/0019-1035(79)90141-6

[CR103] Hasterok D, Halpin JA, Collins AS, et al. (2022) New maps of global geological provinces and tectonic plates. Earth-Sci Rev 231:104069. 10.1016/j.earscirev.2022.104069.

[CR104] Hayashi C, Nakazawa K, Mizuno H (1979) Earth’s melting due to the blanketing effect of the primordial dense atmosphere. Earth Planet Sci Lett 43(1):22–28. 10.1016/0012-821X(79)90152-3

[CR105] Hellard H, Csizmadia Sz, Padovan S, et al. (2019) Retrieval of the fluid Love number k_2_ in exoplanetary transit curves. Astrophys J 878(2):119. 10.3847/1538-4357/ab2048

[CR106] Henrich J (2020) The weirdest people in the West: how the West became psychologically peculiar and particulary prosperous. Penguin Books, London

[CR107] Henrich J, Muthukrishna M (2021) The origins and psychology of human cooperation. Annu Rev Psychol 72(1):207–240. 10.1146/annurev-psych-081920-042106 33006924 10.1146/annurev-psych-081920-042106

[CR108] Herbort O, Sereinig L (2025) The atmospheres of rocky exoplanets: III. Using atmospheric spectra to constrain surface rock composition. Astron Astrophys 699:A67. 10.1051/0004-6361/202453069. arXiv:2505.08883 [astro-ph.EP]

[CR109] Herman J, Huang L, McPeters R, et al. (2018) Interactive comment on “Synoptic ozone, cloud reflectivity, and erythemal irradiance from sunrise to sunset for the whole Earth as viewed by the DSCOVR spacecraft from Lagrange-1” by Jay Herman et al. Atmos Meas Tech 11:177–194. 10.5194/amt-11-177-2018

[CR110] Hinkel NR, Timmes FX, Young PA, et al. (2014) Stellar abundances in the solar neighborhood: the hypatia catalog. Astron J 148(3):54. 10.1088/0004-6256/148/3/54

[CR111] Hirose K, Labrosse S, Hernlund J (2013) Composition and state of the core. Annu Rev Earth Planet Sci 41(1):657–691. 10.1146/annurev-earth-050212-124007

[CR112] Hoffman PF, Li ZX (2009) A palaeogeographic context for Neoproterozoic glaciation. Palaeogeogr Palaeoclimatol Palaeoecol 277(3-4):158–172. 10.1016/j.palaeo.2009.03.013

[CR113] Holland HD (2002) Volcanic gases, black smokers, and the great oxidation event. Geochim Cosmochim Acta 66(21):3811–3826. 10.1016/S0016-7037(02)00950-X

[CR114] Höning D, Spohn T (2016) Continental growth and mantle hydration as intertwined feedback cycles in the thermal evolution of Earth. Phys Earth Planet Inter 255:27–49. 10.1016/j.pepi.2016.03.010

[CR115] Höning D, Spohn T (2023) Land fraction diversity on Earth-like planets and implications for their habitability. Astrobiology 23(4):372–394. 10.1089/ast.2022.0070 36848252 10.1089/ast.2022.0070

[CR116] Höning D, Tosi N, Hansen-Goos H, et al. (2019) Bifurcation in the growth of continental crust. Phys Earth Planet Inter 287:37–50. 10.1016/j.pepi.2019.01.001

[CR117] Höning D, Baumeister P, Grenfell JL, et al. (2021) Early habitability and crustal decarbonation of a stagnant-lid Venus. J Geophys Res Planets 126(10):e2021JE006895. 10.1029/2021JE006895

[CR118] Horner J, Jones B (2008) Jupiter–friend or foe? I: the asteroids. Int J Astrobiol 7(3–4):251–261. 10.1017/S1473550408004187

[CR119] Horner J, Jones BW (2009) Jupiter–friend or foe? II: the centaurs. Int J Astrobiol 8(2):75–80. 10.1017/S1473550408004357

[CR120] Horner J, Jones B, Chambers J (2010) Jupiter–friend or foe? III: the Oort cloud comets. Int J Astrobiol 9(1):1–10. 10.1017/S1473550409990346

[CR121] Hyde WT, Crowley TJ, Baum SK, et al. (2000) Neoproterozoic ‘snowball Earth’ simulations with a coupled climate/ice-sheet model. Nature 405(6785):425–429. 10.1038/35013005 10839531 10.1038/35013005

[CR122] Iess L, Folkner WM, Durante D, et al. (2018) Measurement of Jupiter’s asymmetric gravity field. Nature 555(7695):220–222. 10.1038/nature25776 29517001 10.1038/nature25776

[CR123] Jaupart C, Labrosse S, Lucazeau F, et al. (2015) Temperatures, heat, and energy in the mantle of the Earth. In: Bercovici D, Schubert G (eds) Treatise on geophysics, 2nd edn. vol 7. Elsevier, New York, pp 253–303. 10.1016/B978-0-444-53802-4.00126-3

[CR124] Jellinek A, Lenardic A, Pierrehumbert R (2020) Ice, fire, or fizzle: the climate footprint of Earth’s supercontinental cycles. Geochem Geophys Geosyst 21(2):e2019GC008464. 10.1029/2019GC008464

[CR125] Jiang JH, Zhai AJ, Herman J, et al. (2018) Using deep space climate observatory measurements to study the Earth as an exoplanet. Astron J 156(1):26. 10.3847/1538-3881/aac6e2

[CR126] Jones EM (1985) Where is Everybody: an account of Fermi’s question. https://upload.wikimedia.org/wikipedia/commons/5/54/, retrieved Dec 27, 2025

[CR127] Kálmán Sz, Czismadia Sz, Bernabó LM, et al (2025) Prospects of detecting rotational flatness of exoplanets from space-based photometry. 10.48550/ARXIV.2507.15359

[CR128] Karato S (2011) Rheological structure of the mantle of a super-Earth: some insights from mineral physics. Icarus 212:14–23. 10.1016/j.icarus.2010.12.005

[CR129] Karato S (2015) Water in the evolution of the Earth and other terrestrial planets. In: Stevenson D, Schubert G (eds) Treatise on geophysics, 2nd edn. vol 9. Elsevier, New York, pp 105–144

[CR130] Kasting J (2001) Peter Ward and Donald Brownlee’s “Rare Earth”. Perspect Biol Med 44(1). 10.1353/pbm.2001.0008

[CR131] Kasting J (2025) Oscillating Archean oxygen oases. Nat Geosci 18:372–373. 10.1038/s41561-025-01690-8

[CR132] Kasting JF, Whitmire DP, Reynolds RT (1993) Habitable zones around main sequence stars. Icarus 101(1):108–128. 10.1006/icar.1993.1010 11536936 10.1006/icar.1993.1010

[CR133] Keppler H, Golabek G (2019) Graphite floatation on a magma ocean and the fate of carbon during core formation. Geochem Perspect Lett 11:12–17. 10.7185/geochemlet.1918

[CR134] Kivelson M, Khurana K, Russell C, et al. (1996) Discovery of Ganymede’s magnetic field by the Galileo spacecraft. Nature 384(6609):537–541. 10.1038/384537a0

[CR135] Kleidon A, Lorenz RD (2005) Non-equilibrium thermodynamics and the production of entropy: life, Earth, and beyond. Springer, Berlin 10.1007/b12042

[CR136] Knoll AH, Bambach RK (2000) Directionality in the history of life: diffusion from the left wall or repeated scaling of the right? Paleobiology 26(S4):1–14. 10.1017/S0094837300026865

[CR137] Komacek TD, Kang W, Lustig-Yaeger J, et al. (2021) Constraining the climates of rocky exoplanets. Elements 17(4):251–256. 10.2138/gselements.17.4.251

[CR138] Kopparapu R, Arney G, Haqq-Misra J, et al. (2021) Nitrogen dioxide pollution as a signature of extraterrestrial technology. Astrophys J 908(2):164. 10.3847/1538-4357/abd7f7. arXiv:2102.05027 [astro-ph.EP]

[CR139] Korenaga J (2010) On the likelihood of plate tectonics on super-Earths: does size matter? Astrophys J Lett 725(1):L43. 10.1088/2041-8205/725/1/L43

[CR140] Korenaga J (2021a) Hadean geodynamics and the nature of early continental crust. Precambrian Res 359:106178. 10.1016/j.precamres.2021.106178.

[CR141] Korenaga J (2021b) Was there land on the early Earth? Life 11(11):1142. 10.3390/life11111142 34833018 10.3390/life11111142 PMC8623345

[CR142] Korenaga J, Planavsky NJ, Evans DA (2017) Global water cycle and the coevolution of the Earth’s interior and surface environment. Philos Trans R Soc A, Math Phys Eng Sci 375(2094):20150393. 10.1098/rsta.2015.0393 10.1098/rsta.2015.0393 PMC5394256 28416728

[CR143] Kreidberg L, Koll DDB, Morley C, et al. (2019) Absence of a thick atmosphere on the terrestrial exoplanet LHS 3844b. Nature 573(7772):87–90. 10.1038/s41586-019-1497-4. arXiv:1908.06834 [astro-ph.EP] 31427764 10.1038/s41586-019-1497-4

[CR144] Krissansen-Totton J, Arney GN, Catling DC (2018) Constraining the climate and ocean pH of the early Earth with a geological carbon cycle model. Proc Natl Acad Sci USA 115(16):4105–4110. 10.1073/pnas.1721296115 29610313 10.1073/pnas.1721296115 PMC5910859

[CR145] Kubyshkina D, Way M, Dandouras I, et al (2026) Upper atmosphere dynamics and drivers of volatiles loss from terrestrial-type (exo)planets. Space Sci Rev 222 10.1007/s11214-026-01283-w PMC12999714 41869552

[CR146] Kuhn WR, Atreya SK (1979) Ammonia photolysis and the greenhouse effect in the primordial atmosphere of the Earth. Icarus 37(1):207–213. 10.1016/0019-1035(79)90126-X

[CR147] Lagage P-O, Mandell A, Giménez A, et al (2026) The future of rocky worlds exploration. Space Sci Rev 222

[CR148] Lammer H, Bredehöft J, Coustenis A, et al. (2009) What makes a planet habitable? Astron Astrophys Rev 17(2):181–249. 10.1007/s00159-009-0019-z

[CR149] Lammer H, Scherf M, Spross L (2024) Eta-Earth revisited I: deriving a formula for Earth-like habitats. Astrobiology 24(10):e897–e915. 10.1089/ast.2023.007 10.1089/ast.2023.0075 39481024

[CR150] Lan Z, Huyskens MH, Le Hir G, et al. (2022) Massive volcanism may have foreshortened the Marinoan snowball Earth. Geophys Res Lett 49(6):e2021GL097156. 10.1029/2021GL097156

[CR151] Landman R, Stolker T, Snellen IAG, et al. (2024) Pictoris b through the eyes of the upgraded CRIRES+. Atmospheric composition, spin rotation, and radial velocity. Astron Astrophys 682:A48. 10.1051/0004-6361/202347846. arXiv:2311.13527 [astro-ph.EP]

[CR152] Langmuir CH, Broeker W (2012) How to build a habitable planet. Princeton University Press, Princeton

[CR153] Laskar J, Joutel F, Robutel P (1993) Stabilization of the Earth’s obliquity by the Moon. Nature 361(6413):615–617. 10.1038/361615a0

[CR154] Lebrun T, Massol H, Chassefière E, et al. (2013) Thermal evolution of an early magma ocean in interaction with the atmosphere. J Geophys Res Planets 118(6):1155–1176. 10.1002/jgre.20068

[CR155] Lecavelier des Etangs A, Sirothia SK, Gopal-Krishna, et al. (2013) Hint of 150 MHz radio emission from the Neptune-mass extrasolar transiting planet HAT-P-11b. Astron Astrophys 552:A65. 10.1051/0004-6361/201219789. arXiv:1302.4612 [astro-ph.EP]

[CR156] Lee CTA, Jiang H, Dasgupta R, et al. (2019) A framework for understanding whole-Earth carbon cycling. In: Orecutt BN, Daniel I, Dasgupta R (eds) Deep carbon: past to present. Cambridge University Press, Cambridge, pp 313–357

[CR157] Lerner P, Romanou A, Way M, et al. (2025) Obliquity dependence of ocean productivity and atmospheric CO_2_ on Earth-like worlds. Astrophys J 979(2):234. 10.3847/1538-4357/ada277

[CR158] Lineweaver CH (2025) Galactic habitability. Oxford research encyclopedia of planetary science. Oxford University Press, Oxford. 10.1093/acrefore/9780190647926.013.305

[CR159] Lineweaver CH, Chopra A (2012) The habitability of our Earth and other Earths: astrophysical, geochemical, geophysical, and biological limits on planet habitability. Annu Rev Earth Planet Sci 40(1):597–623. 10.1146/annurev-earth-042711-105531

[CR160] Lineweaver CH, Chopra A (2025) Other Earths, Gaian bottlenecks and Darwinized Gaias. Philos Trans R Soc, Biol Sci 380:20240438. 10.1098/rstb.2024.0438 10.1098/rstb.2024.0438 PMC12329459 40770977

[CR161] Lingam M, Balbi A (2024) From stars to life - a quantitative approach to astrobiology. Cambridge University Press, Cambridge

[CR162] Lingam M, Loeb A (2019a) Dependence of biological activity on the surface water fraction of planets. Astron J 157(1):1–12. 10.3847/1538-3881/aaf420

[CR163] Lingam M, Loeb A (2019b) Physical constraints for the evolution of life on exoplanets. Rev Mod Phys 91(2):021002. 10.1103/RevModPhys.91.021002

[CR164] Lingam M, Loeb A (2021) Life in the cosmos: from biosignatures to technosignatures. Harvard University Press, Cambridge

[CR165] Livengood TA, Deming LD, A’Hearn MF, et al. (2011) Properties of an Earth-like planet orbiting a Sun-like star: Earth observed by the EPOXI mission. Astrobiology 11(9):907–930. 10.1089/ast.2011.0614 22077375 10.1089/ast.2011.0614

[CR166] Loeb A, Turner EL (2012) Detection technique for artificially illuminated objects in the outer Solar System and beyond. Astrobiology 12(4):290–294. 10.1089/ast.2011.0758. arXiv:1110.6181 [astro-ph.EP] 22490065 10.1089/ast.2011.0758 PMC3330268

[CR167] Lognonné P, Johnson CL (2015) Planetary seismology. In: Spohn T, Schubert G (eds) Treatise on geophysics, 2nd edn. vol 10. Elsevier, New York, pp 65–120

[CR168] Lognonné P, Banerdt WB, Giardini D, et al. (2019) SEIS: Insight’s Seismic Experiment for Internal Structure of Mars. Space Sci Rev 215(1):12. 10.1007/s11214-018-0574-6 30880848 10.1007/s11214-018-0574-6 PMC6394762

[CR169] Lorenz EN (1995) The essence of chaos. University of Washington Press, Seattle

[CR170] Lourenço DL, Rozel AB, Gerya T, et al. (2018) Efficient cooling of rocky planets by intrusive magmatism. Nat Geosci 11(5):322–327. 10.1038/s41561-018-0094-8

[CR171] Lourenço DL, Rozel AB, Ballmer MD, et al. (2020) Plutonic-squishy lid: a new global tectonic regime generated by intrusive magmatism on Earth-like planets. Geochem Geophys Geosyst 21(4):e2019GC008756. 10.1029/2019GC008756

[CR172] Lourenço DL, Breuer D, Arnould M, et al (2026) Rocky planets as heat engines. Space Sci Rev 222 10.1007/s11214-026-01317-3 PMC13503420 42643347

[CR173] Love AEH (1911) Some problems of geodynamics: being an essay to which the Adams prize in the university of Cambridge was adjudged in 1911. University Press, Cambridge

[CR174] Lustig-Yaeger J, Diamond-Lowe H, Guimond CM, et al (2026) The geoscience and observables of hot rocky exoplanets. Space Sci Rev 222

[CR175] LUVOIR Team (2019) The LUVOIR mission concept study final report. arXiv preprint. arXiv:1912.06219

[CR176] Lyons TW, Reinhard CT, Planavsky NJ (2014) The rise of oxygen in Earth’s early ocean and atmosphere. Nature 506(7488):307–315. 10.1038/nature13068 24553238 10.1038/nature13068

[CR177] Lyons TW, Diamond CW, Planavsky NJ, et al. (2021) Oxygenation, life, and the planetary system during Earth’s middle history: an overview. Astrobiology 21(8):906–923. 10.1089/ast.2020.2418 34314605 10.1089/ast.2020.2418 PMC8403206

[CR178] Margot JL, Campbell DB, Giorgini JD, et al. (2021) Spin state and moment of inertia of Venus. Nat Astron 5(7):676–683. 10.1038/s41550-021-01339-7

[CR179] Marty B (2012) The origins and concentrations of water, carbon, nitrogen and noble gases on Earth. Earth Planet Sci Lett 313:56–66. 10.1016/j.epsl.2011.10.040

[CR180] Marty B, Zimmermann L, Pujol M, et al. (2013) Nitrogen isotopic composition and density of the Archean atmosphere. Science 342(6154):101–104. 10.1126/science.1240971 24051244 10.1126/science.1240971

[CR181] Marty B, Avice G, Bekaert DV, et al. (2018) Salinity of the Archaean oceans from analysis of fluid inclusions in quartz. C R Géosci 350(4):154–163. 10.1016/j.crte.2017.12.002

[CR182] Maurice M, Dasgupta R, Hassanzadeh P (2024) Volatile atmospheres of lava worlds. Astron Astrophys 688:A47. 10.1051/0004-6361/202347749

[CR183] McIntyre SR, Lineweaver CH, Ireland MJ (2019) Planetary magnetism as a parameter in exoplanet habitability. Mon Not R Astron Soc 485(3):3999–4012. 10.1093/mnras/stz667

[CR184] Meadows VS, Reinhard CT, Arney GN, et al. (2018) Exoplanet biosignatures: understanding oxygen as a biosignature in the context of its environment. Astrobiology 18(6):630–662. 10.1089/ast.2017.1727 29746149 10.1089/ast.2017.1727 PMC6014580

[CR185] Meadows V, Graham H, Abrahamsson V, et al (2022) Community Report from the Biosignatures Standards of Evidence Workshop. arXiv e-prints. arXiv:2210.14293

[CR186] Miozzi F, Morard G, Antonangeli D, et al. (2018) Equation of state of SiC at extreme conditions: new insight into the interior of carbon-rich exoplanets. J Geophys Res Planets 123(9):2295–2309. 10.1029/2018JE005582

[CR187] Mizuno H, Nakazawa K, Hayashi C (1978) Instability of a gaseous envelope surrounding a planetary core and formation of giant planets. Prog Theor Phys 60(3):699–710. 10.1143/PTP.60.699

[CR188] Mojzsis S, Mark Harrison T, Pidgeon R (2001) Oxygen-isotope evidence from ancient zircons for liquid water at the Earth’s surface 4300 myr ago. Nature 409:178–181. 10.1038/35051557 11196638 10.1038/35051557

[CR189] Moody ER, Álvarez-Carretero S, Mahendrarajah TA, et al. (2024) The nature of the last universal common ancestor and its impact on the early Earth system. Nat Ecol Evol 8(9):1654–1666. 10.1038/s41559-024-02461-1 38997462 10.1038/s41559-024-02461-1 PMC11383801

[CR190] Moore WB, Webb AAG (2013) Heat-pipe Earth. Nature 501(7468):501–505. 10.1038/nature12473 24067709 10.1038/nature12473

[CR191] Moore WB, Simon JI, Webb AAG (2017) Heat-pipe planets. Earth Planet Sci Lett 474:13–19

[CR192] Morgan JV, Bralower TJ, Brugger J, et al. (2022) The Chicxulub impact and its environmental consequences. Nat Rev, Earth Environ 3(5):338–354. 10.1038/s43017-022-00283-y

[CR193] Morris SC (2003) Life’s solution: inevitable humans in a lonely universe. Cambridge University Press, Cambridge

[CR194] Morris SC (2011) Predicting what extra-terrestrials will be like: and preparing for the worst. Philos Trans R Soc A, Math Phys Eng Sci 369(1936):555–571. 10.1098/rsta.2010.0276 10.1098/rsta.2010.0276 21220280

[CR195] Mouser MD, Dygert N, Anzures BA, et al. (2021) Experimental investigation of Mercury’s magma ocean viscosity: implications for the formation of Mercury’s cumulate mantle, its subsequent dynamic evolution, and crustal petrogenesis. J Geophys Res Planets 126(11):e2021JE006946. 10.1029/2021JE006946

[CR196] Mukherjee I, Corkrey R, Gregory D, et al. (2025) A billion years of geological drama–boring or brilliant? Gondwana Res 142:1–19. 10.1016/j.gr.2025.02.018

[CR197] Neumann W, Breuer D, Spohn T (2013) The thermo-chemical evolution of asteroid 21 Lutetia. Icarus 224(1):126–143. 10.1016/j.icarus.2013.02.025

[CR198] Nicholls H, Lichtenberg T, Bower DJ, et al. (2024) Magma ocean evolution at arbitrary redox state. J Geophys Res Planets 129(12):e2024JE008576. 10.1029/2024JE008576 10.1029/2024JE008576 PMC11667094 39722853

[CR199] Nisr C, Meng Y, MacDowell A, et al. (2017) Thermal expansion of SiC at high pressure-temperature and implications for thermal convection in the deep interiors of carbide exoplanets. J Geophys Res Planets 122(1):124–133. 10.1002/2016JE005158

[CR200] Noack L, Breuer D (2014) Plate tectonics on rocky exoplanets: influence of initial conditions and mantle rheology. Planet Space Sci 98:41–49. 10.1016/j.pss.2013.06.020

[CR201] Olson S, Jansen MF, Abbot DS, et al. (2022) The effect of ocean salinity on climate and its implications for Earth’s habitability. Geophys Res Lett 49(10):e2021GL095748. 10.1029/2021GL095748 10.1029/2021GL095748 PMC9286645 35864818

[CR202] O’Neill C, Jellinek A, Lenardic A (2007) Conditions for the onset of plate tectonics on terrestrial planets and moons. Earth Planet Sci Lett 261(1–2):20–32. 10.1016/j.epsl.2007.05.038

[CR203] O’Rourke J, Buz J, Fu R, et al. (2019) Detectability of remanent magnetism in the crust of Venus. Geophys Res Lett 46(11):5768–5777. 10.1029/2019GL082725

[CR204] Ouyang S, Ji J, Li C, et al. (2025) Phanerozoic onset of massive continental weathering. Geophys Res Lett 52(17):e2025GL117072. 10.1029/2025GL117072

[CR205] Padovan S, Spohn T, Baumeister P, et al. (2018) Matrix-propagator approach to compute fluid love numbers and applicability to extrasolar planets. Astron Astrophys 620:A178. 10.1051/0004-6361/201834181

[CR206] Palme H, Lodders K, Jones A (2014) Solar System abundances of the elements. In: Holland H, Turekian K (eds) Treatise on geochemistry, 2nd edn. vol 2. Elsevier, New York, pp 15–36. 10.1016/B978-0-08-095975-7.00118-2

[CR207] Parnell J, Brolly C (2021) Increased biomass and carbon burial 2 billion years ago triggered mountain building. Commun Earth Environ 2(238). 10.1038/s43247-021-00313-5

[CR208] Pellissier L, Heine C, Rosauer DF, et al. (2018) Are global hotspots of endemic richness shaped by plate tectonics? Biol J Linn Soc 123(1):247–261. 10.1093/biolinnean/blx125

[CR209] Peña-Moñino L, Pérez-Torres M, Varela J, et al. (2024) Magnetohydrodynamic simulations of the space weather in Proxima b: habitability conditions and radio emission. Astron Astrophys 688:A138. 10.1051/0004-6361/202349042

[CR210] Peplowski PN, Klima RL, Lawrence DJ, et al. (2016) Remote sensing evidence for an ancient carbon-bearing crust on Mercury. Nat Geosci 9(4):273–276. 10.1038/ngeo2669

[CR211] Piaulet C, Benneke B, Almenara JM, et al. (2023) Evidence for the volatile-rich composition of a 1.5-Earth-radius planet. Nat Astron 7(2):206–222. 10.1038/s41550-022-01835-4

[CR212] Piaulet-Ghorayeb C, Benneke B, Turbet M, et al. (2025) Strict limits on potential secondary atmospheres on the temperate rocky exo-Earth TRAPPIST-1d. Astrophys J 989(2):181. 10.3847/1538-4357/adf207

[CR213] Pommier A, Tauber M, Pirotte H, et al. (2023) Experimental investigation of the bonding of sulfur in highly reduced silicate glasses and melts. Geochim Cosmochim Acta 363:114–128. 10.1016/j.gca.2023.10.027

[CR214] Powell R (2020) Contingency and convergence: toward a cosmic biology of body and mind, vol 25. MIT Press, Cambridge

[CR215] Putirka KD, Dorn C, Hinkel NR, et al. (2021) Compositional diversity of rocky exoplanets. Elements 17(4):235–240. 10.2138/gselements.17.4.235

[CR216] Quanz SP, Ottiger M, Fontanet E, et al. (2022) Large interferometer for exoplanets (LIFE): I. Improved exoplanet detection yield estimates for a large mid-infrared space-interferometer mission. Astron Astrophys 664:A21. 10.1051/0004-6361/202140366

[CR217] Rackham BV, Espinoza N, Berdyugina SV, et al. (2023) The effect of stellar contamination on low-resolution transmission spectroscopy: needs identified by NASA’s exoplanet exploration program study analysis group 21. RAS Tech Instrum 2(1):148–206. 10.1093/rasti/rzad009. arXiv:2201.09905 [astro-ph.IM]

[CR218] Ramstad R, Barabash S (2021) Do intrinsic magnetic fields protect planetary atmospheres from stellar winds? Space Sci Rev 217. 10.1007/s11214-021-00791-1

[CR219] Rauer H, Catala C, Aerts C, et al. (2014) The PLATO 2.0 mission. Exp Astron 38:249–330. 10.1007/s10686-014-9383-4

[CR220] Reinhard CT, Planavsky NJ, Olson SL, et al. (2016) Earth’s oxygen cycle and the evolution of animal life. Proc Natl Acad Sci USA 113(32):8933–8938. 10.1073/pnas.1521544113 27457943 10.1073/pnas.1521544113 PMC4987840

[CR221] Retallack GJ (2015) Coevolution of life and Earth. In: Stevenson D, Schubert G (eds) Treatise on geophysics, 2nd edn. vol 9. Elsevier, New York, pp 281–302

[CR222] Rey PF, Coltice N, Flament N (2024) Archean geodynamics underneath weak, flat, and flooded continents. Elements 20(3):180–186. 10.2138/gselements.20.3.180

[CR223] Richey-Yowell T, Shkolnik EL, Llama J, et al. (2025) Stringent limits on H3+ emission from the hot jupiters WASP-80b and WASP-69b. Astron J 169(6):327. 10.3847/1538-3881/adce76

[CR224] Rimmer PB, Ranjan S, Rugheimer S (2021) Life’s origins and the search for life on rocky exoplanets. Elements 17(4):265–270. 10.2138/gselements.17.4.265

[CR225] Ringwood AE (1961) Changes in solar luminosity and some possible terrestrial consequences. Geochim Cosmochim Acta 21(3–4):295–296. 10.1016/S0016-7037(61)80064-1

[CR226] Robinson TD, Meadows VS, Crisp D, et al. (2011) Earth as an extrasolar planet: Earth model validation using EPOXI Earth observations. Astrobiology 11(5):393–408. 10.1089/ast.2011.0642 21631250 10.1089/ast.2011.0642 PMC3133830

[CR227] Rolf T, Weller M, Gülcher A, et al. (2022) Dynamics and evolution of Venus’ mantle through time. Space Sci Rev 218(8):70. 10.1007/s11214-022-00937-9

[CR228] Rosing MT, Bird DK, Sleep NH, et al. (2006) The rise of continents—an essay on the geologic consequences of photosynthesis. Palaeogeogr Palaeoclimatol Palaeoecol 232(2):99–113. 10.1016/j.palaeo.2006.01.007

[CR229] Rosing MT, Bird DK, Sleep NH, et al. (2010) No climate paradox under the faint early Sun. Nature 464(7289):744–747. 10.1038/nature08955 20360739 10.1038/nature08955

[CR230] Sagan C, Mullen G (1972) Earth and Mars: evolution of atmospheres and surface temperatures. Science 177(4043):52–56. 10.1126/science.177.4043.52 17756316 10.1126/science.177.4043.52

[CR231] Salvador A, Massol H, Davaille A, et al. (2017) The relative influence of H_2_O and CO_2_ on the primitive surface conditions and evolution of rocky planets. J Geophys Res Planets 122(7):1458–1486. 10.1002/2017JE005286

[CR232] Salvador A, Avice G, Breuer D, et al. (2023) Magma ocean, water, and the early atmosphere of Venus. Space Sci Rev 219(7):51. 10.1007/s11214-023-00995-7

[CR233] Schaefer LK, Parmentier V (2021) The air over there: exploring exoplanet atmospheres. Elements 17(4):257–263. 10.2138/gselements.17.4.257

[CR234] Scherf M, Lammer H, Spross L (2024) Eta-Earth revisited ii: deriving a maximum number of Earth-like habitats in the galactic disk. Astrobiology 24(10):e916–e1061. 10.1089/ast.2023.0076 39481023 10.1089/ast.2023.0076

[CR235] Schmidt MW, Gao C, Golubkova A, et al. (2014) Natural moissanite (SiC)–a low temperature mineral formed from highly fractionated ultra-reducing COH-fluids. Prog Earth Planet Sci 1(1):27

[CR236] Schoene B, Eddy MP, Samperton KM, et al. (2019) U-Pb constraints on pulsed eruption of the Deccan Traps across the end-Cretaceous mass extinction. Science 363(6429):862–866 30792300 10.1126/science.aau2422

[CR237] Schubert G, Turcotte DL, Olson P (2001) Mantle convection in the Earth and planets. Cambridge University Press, Cambridge. 10.1017/CBO9780511612879

[CR238] Schulze-Makuch D, Irwin LN (2018) Life in the universe: expectations and constraints, 3rd edn. Springer, Berlin. 10.1007/978-3-319-97658-7

[CR239] Schwieterman EW, Kiang NY, Parenteau MN, et al. (2018) Exoplanet biosignatures: a review of remotely detectable signs of life. Astrobiology 18(6):663–708. 10.1089/ast.2017.17 29727196 10.1089/ast.2017.1729 PMC6016574

[CR240] Shorttle O, Hinkel NR, Unterborn CT (2021) Why geosciences and exoplanetary sciences need each other. Elements 17(4):229–234. 10.2138/gselements.17.4.229

[CR241] Simpson F (2017) Bayesian evidence for the prevalence of waterworlds. Mon Not R Astron Soc 468:2803–2815. 10.1093/mnras/stx516

[CR242] Sleep NH (2015) Evolution of the Earth: plate tectonics through time. In: Stevenson D, Schubert G (eds) Treatise on geophysics, 2nd edn. vol 9. Elsevier, New York, pp 145–172

[CR243] Sleep NH, Lowe DR (2014) Physics of crustal fracturing and chert dike formation triggered by asteroid impact, 3.26 Ga, Barberton greenstone belt, South Africa. Geochem Geophys Geosyst 15(4):1054–1070. 10.1002/2014GC005229

[CR244] Smith JM, Szathmáry E (1995) The major transitions in evolution. Oxford University Press, Oxford

[CR245] Sohl F, Schubert G (2015) Interior structure, composition, and mineralogy of the terrestrial planets. In: Spohn T, Schubert G (eds) Treatise on geophysics, 2nd edn. vol 10. Elsevier, New York, pp 23–64

[CR246] Som SM, Catling DC, Harnmeijer JP, et al. (2012) Air density 2.7 billion years ago limited to less than twice modern levels by fossil raindrop imprints. Nature 484(7394):359–362. 10.1038/nature10890 22456703 10.1038/nature10890

[CR247] Som SM, Buick R, Hagadorn JW, et al. (2016) Earth’s air pressure 2.7 billion years ago constrained to less than half of modern levels. Nat Geosci 9(6):448–451. 10.1038/ngeo2713

[CR248] Southam G, Westall F, Spohn T (2015) Geology, life and habitability. In: Spohn T, Schubert G (eds) Treatise on geophysics, 2nd edn. vol 10. Elsevier, New York, pp 473–486

[CR249] Spaargaren RJ, Wang HS, Mojzsis SJ, et al. (2023) Plausible constraints on the range of bulk terrestrial exoplanet compositions in the solar neighborhood. Astrophys J 948(1):53

[CR250] Spencer CJ (2022) Biogeodynamics: coupled evolution of the biosphere, atmosphere, and lithosphere. Geology 50(8):867–868. 10.1130/GEOL50THAUG.1

[CR251] Sprain CJ, Renne PR, Vanderkluysen L, et al. (2019) The eruptive tempo of Deccan volcanism in relation to the Cretaceous-Paleogene boundary. Science 363(6429):866–870. 10.1126/science.aav1446 30792301 10.1126/science.aav1446

[CR252] Stamenković V, Breuer D, Spohn T (2011) Thermal and transport properties of mantle rock at high pressure: applications to super-Earths. Icarus 216(2):572–596. 10.1016/j.icarus.2011.09.030

[CR253] Stamenković V, Noack L, Breuer D, et al. (2012) The influence of pressure-dependent viscosity on the thermal evolution of super-Earths. Astrophys J 748(41):41. 10.1088/0004-637X/748/1/41

[CR254] Steinmeyer ML, Noack L, Baumeister P, et al (2026) Evolution and observable properties of rocky planet atmospheres. Space Sci Rev 222 10.1007/s11214-026-01308-4 PMC13350138 42434139

[CR255] Stern RJ (2016) Is plate tectonics needed to evolve technological species on exoplanets? Geosci Front 7(4):573–580. 10.1016/j.gsf.2015.12.002

[CR256] Stern RJ, Gerya TV (2024) The importance of continents, oceans and plate tectonics for the evolution of complex life: implications for finding extraterrestrial civilizations. Sci Rep 14:8552. 10.1038/s41598-024-54700-x 38609425 10.1038/s41598-024-54700-x PMC11015018

[CR257] Stevenson DJ, Spohn T, Schubert G (1983) Magnetism and thermal evolution of the terrestrial planets. Icarus 54(3):466–489. 10.1016/0019-1035(83)90241-5

[CR258] Stüeken EE, Kipp MA, Koehler MC, et al. (2016) The evolution of Earth’s biogeochemical nitrogen cycle. Earth-Sci Rev 160:220–239. 10.1016/j.earscirev.2016.07.007

[CR259] Szathmáry E (2015) Toward major evolutionary transitions theory 2.0. Proc Natl Acad Sci USA 112(33):10104–10111 25838283 10.1073/pnas.1421398112 PMC4547294

[CR260] Szathmáry E, Smith JM (1995) The major evolutionary transitions. Nature 374(6519):227–232. 10.1038/374227a0 7885442 10.1038/374227a0

[CR261] Tarter JC, Duquet RT, Clark TA, et al. (1983) Recent SETI observations at Arecibo. Acta Astronaut 10:277–282. 10.1016/0094-5765(83)90077-2 11541557 10.1016/0094-5765(83)90077-2

[CR262] Tastassa AC, Sharaby Y, Lang-Yona N (2024) Aeromicrobiology: a global review of the cycling and relationships of bioaerosols with the atmosphere. Sci Total Environ 912:168478 37967625 10.1016/j.scitotenv.2023.168478

[CR263] Taylor SR (1998) Destiny or chance: our Solar System and its place in the cosmos. Cambridge University Press, Cambridge

[CR264] Trifonov T (2026) Radial velocity technique. In: Mandel I (ed) Encyclopedia of astrophysics. Elsevier, Amsterdam, pp 320–341. 10.1016/B978-0-443-21439-4.00022-5

[CR265] Turner JD, Zarka P, Grießmeier JM, et al. (2021) The search for radio emission from the exoplanetary systems 55 Cancri, Andromedae, and Boötis using LOFAR beam-formed observations. Astron Astrophys 645:A59. 10.1051/0004-6361/201937201. arXiv:2012.07926 [astro-ph.EP]

[CR266] Turyshev SG (2025) Direct high-resolution imaging of Earth-like exoplanets. arXiv:2506.20236

[CR267] Unterborn CT, Kabbes JE, Pigott JS, et al. (2014) The role of carbon in extrasolar planetary geodynamics and habitability. Astrophys J 793(2):124. 10.1088/0004-637X/793/2/124

[CR268] Unterborn CT, Desch SJ, Hinkel NR, et al. (2018) Inward migration of the TRAPPIST-1 planets as inferred from their water-rich compositions. Nat Astron 2(4):297–302. 10.1038/s41550-018-0411-6

[CR269] Unterborn CT, Byrne PK, Anbar AD, et al (2020) Exogeoscience and its role in characterizing exoplanet habitability and the detectability of life. arXiv:2007.08665

[CR270] Valley JW, Peck WH, King EM, et al. (2002) A cool early Earth. Geology 30(4):351–354. 10.1130/0091-7613(2002)030<0351:ACEE>2.0.CO;2

[CR271] van Hoolst T, Noack L, Rivoldini A (2019) Exoplanet interiors and habitability. Adv Phys X 4(1):1630316. 10.1080/23746149.2019.1630316

[CR272] Van Looveren G, Boro Saikia S, Herbort O, et al. (2025) Habitable Zone and Atmosphere Retention Distance (HaZARD): stellar-evolution-dependent loss models of secondary atmospheres. Astron Astrophys 694:A310. 10.1051/0004-6361/202452998

[CR273] Vidotto AA, Feeney N, Groh JH (2019) Can we detect aurora in exoplanets orbiting M dwarfs? Mon Not R Astron Soc 488(1):633–644. 10.1093/mnras/stz1696

[CR274] Walker J, Hayes P, Kasting J (1981) A negative feedback mechanism for the long-term stabilization of Earth’s surface temperature. J Geophys Res 86:9776–9782. 10.1029/JC086iC10p09776

[CR275] Waltham D (2014) Lucky planet: why Earth is exceptional - and what that means for life in the universe. Basic Books, NY

[CR276] Wang HS, Lineweaver CH, Quanz SP, et al. (2022) A model Earth-sized planet in the habitable zone of Centauri A/B. Astrophys J 927(2):134. 10.3847/1538-4357/ac4e8c

[CR277] Ward P (2007) Life as we do not know it. The NASA search for (and synthesis of) alien life. Penguin, NY

[CR278] Ward P, Brownlee D (2000) Rare Earth: why complex life is uncommon in the universe. Springer, Heidelberg

[CR279] Way MJ, Del Genio AD (2020) Venusian habitable climate scenarios: modeling Venus through time and applications to slowly rotating Venus-like exoplanets. J Geophys Res Planets 125(5):e2019JE006276. 10.1029/2019JE006276

[CR280] Way MJ, Davies HS, Duarte JC, et al. (2021) The climates of Earth’s next supercontinent: effects of tectonics, rotation rate, and insolation. Geochem Geophys Geosyst 22(8):e2021GC009983. 10.1029/2021GC009983

[CR281] Way MJ, Ernst RE, Scargle JD (2022) Large-scale volcanism and the heat death of terrestrial worlds. Planet Sci J 3(4):92. 10.3847/PSJ/ac6033

[CR282] Way MJ, Ostberg C, Foley BJ, et al (2023) Synergies between Venus & exoplanetary observations. Space Sci Rev 219(13). 10.1007/s11214-023-00953-3 10.1007/s11214-023-00953-3 PMC9911515 36785654

[CR283] Webb S (2015) If the universe is teeming with aliens... Where is everybody?: Seventy-five solutions to the Fermi paradox and the problem of extraterrestrial life. Springer, Cham. 10.1007/978-3-319-13236-5

[CR284] Wetherill GW (1994) Possible consequences of absence of “Jupiters” in planetary systems. Astrophys Space Sci 212(1):23–32. 10.1007/BF00984505 11539457 10.1007/BF00984505

[CR285] Wignall PB (2001) Large igneous provinces and mass extinctions. Earth-Sci Rev 53(1–2):1–33. 10.1016/S0012-8252(00)00037-4

[CR286] Wilde SA, Valley JW, Peck WH, et al. (2001) Evidence from detrital zircons for the existence of continental crust and oceans on the Earth 4.4 (gyr) ago. Nature 409(6817):175–178. 10.1038/35051550 11196637 10.1038/35051550

[CR287] Wolfe JM, Fournier GP (2018) Horizontal gene transfer constrains the timing of methanogen evolution. Nat Ecol Evol 2(5):897–903. 10.1038/s41559-018-0513-7 29610466 10.1038/s41559-018-0513-7

[CR288] Wordsworth RD (2016) The climate of early Mars. Annu Rev Earth Planet Sci 44(1):381–408

[CR289] Wright JT, Griffith RL, Sigurdsson S, et al. (2014) The Ĝ infrared search for extraterrestrial civilizations with large energy supplies. II. Framework, strategy, and first result. Astrophys J 792(1):27. 10.1088/0004-637X/792/1/27

[CR290] Xu S, Bonsor A (2021) Exogeology from polluted white dwarfs. Elements 17(4):241–244. 10.2138/gselements.17.4.241

[CR291] Zahnle KJ, Catling DC (2017) The cosmic shoreline: the evidence that escape determines which planets have atmospheres, and what this may mean for Proxima Centauri B. Astrophys J 843(2):122. 10.3847/1538-4357/aa7846

[CR292] Zieba S, Kreidberg L, Ducrot E, et al. (2023) No thick carbon dioxide atmosphere on the rocky exoplanet TRAPPIST-1c. Nature 620(7975):746–749. 10.1038/s41586-023-06232-z. arXiv:2306.10150 [astro-ph.EP] 37337068 10.1038/s41586-023-06232-z PMC10447244

[CR293] Zuluaga JI, Salazar JF, Cuartas-Restrepo P, et al. (2014) The habitable zone of inhabited planets. Biogeosci Discuss 11(6):8443–8483. 10.5194/bgd-11-8443-2014

[CR294] Zurkowski CC, Yang J, Chariton S, et al. (2022) Synthesis and stability of an eight-coordinated Fe_3_O_4_ high-pressure phase: implications for the mantle structure of super-Earths. J Geophys Res Planets 127(8):e2022JE007344. 10.1029/2022JE007344

